# Implementation of CRISPR/Cas9 Genome Editing to Generate Murine Lung Cancer Models That Depict the Mutational Landscape of Human Disease

**DOI:** 10.3389/fcell.2021.641618

**Published:** 2021-03-02

**Authors:** Oliver Hartmann, Michaela Reissland, Carina R. Maier, Thomas Fischer, Cristian Prieto-Garcia, Apoorva Baluapuri, Jessica Schwarz, Werner Schmitz, Martin Garrido-Rodriguez, Nikolett Pahor, Clare C. Davies, Florian Bassermann, Amir Orian, Elmar Wolf, Almut Schulze, Marco A. Calzado, Mathias T. Rosenfeldt, Markus E. Diefenbacher

**Affiliations:** ^1^Deregulated Protein Stability and Cancer Laboratory, Lehrstuhl für Biochemie und Molekularbiologie, Biozentrum, Universität Würzburg, Würzburg, Germany; ^2^Mildred Scheel Early Career Center, Würzburg, Germany; ^3^Tumour Metabolism and Microenvironment Group, DKFZ Heidelberg, Heidelberg, Germany; ^4^Klinik und Poliklinik für Strahlentherapie, Universitätsklinikum Würzburg, Würzburg, Germany; ^5^Faculty of Medicine, TICC, Technion Haifa, Haifa, Israel; ^6^Cancer Systems Biology Group, Lehrstuhl für Biochemie und Molekularbiologie, Biozentrum, Universität Würzburg, Würzburg, Germany; ^7^Lehrstuhl für Biochemie und Molekularbiologie, Biozentrum, Universität Würzburg, Würzburg, Germany; ^8^Instituto Maimónides de Investigación Biomédica de Córdoba (IMIBIC), Córdoba, Spain; ^9^Departamento de Biología Celular, Fisiología e Inmunología, Universidad de Córdoba, Córdoba, Spain; ^10^Hospital Universitario Reina Sofía, Córdoba, Spain; ^11^Institute of Cancer and Genomic Sciences, College of Medical and Dental Sciences, University of Birmingham, Birmingham, United Kingdom; ^12^Department of Medicine III, Klinikum rechts der Isar, Technical University of Munich, Munich, Germany; ^13^TranslaTUM, Center for Translational Cancer Research, Technical University of Munich, Munich, Germany; ^14^Institut für Pathologie, Universitätsklinikum Würzburg, Würzburg, Germany

**Keywords:** non-small cell lung cancer, CRISPR-Cas9, mouse model, lung cancer, MYC, JUN, *KRAS*, *TP53*

## Abstract

Lung cancer is the most common cancer worldwide and the leading cause of cancer-related deaths in both men and women. Despite the development of novel therapeutic interventions, the 5-year survival rate for non-small cell lung cancer (NSCLC) patients remains low, demonstrating the necessity for novel treatments. One strategy to improve translational research is the development of surrogate models reflecting somatic mutations identified in lung cancer patients as these impact treatment responses. With the advent of CRISPR-mediated genome editing, gene deletion as well as site-directed integration of point mutations enabled us to model human malignancies in more detail than ever before. Here, we report that by using CRISPR/Cas9-mediated targeting of *Trp53* and *KRas*, we recapitulated the classic murine NSCLC model *Trp53^*fl/fl*^:lsl-KRas^*G12D/wt*^*. Developing tumors were indistinguishable from *Trp53^*fl/fl*^:lsl-KRas^*G12D/wt*^*-derived tumors with regard to morphology, marker expression, and transcriptional profiles. We demonstrate the applicability of CRISPR for tumor modeling *in vivo* and ameliorating the need to use conventional genetically engineered mouse models. Furthermore, tumor onset was not only achieved in constitutive *Cas9* expression but also in wild-type animals *via* infection of lung epithelial cells with two discrete AAVs encoding different parts of the CRISPR machinery. While conventional mouse models require extensive husbandry to integrate new genetic features allowing for gene targeting, basic molecular methods suffice to inflict the desired genetic alterations *in vivo*. Utilizing the CRISPR toolbox, *in vivo* cancer research and modeling is rapidly evolving and enables researchers to swiftly develop new, clinically relevant surrogate models for translational research.

## Introduction

Lung cancer is the leading cause of cancer-related deaths in the Western world, claiming around 1.8 million lives every year (Bray et al., 2018). While novel treatment options such as immune checkpoint inhibitors or receptor tyrosine kinase inhibitors (RTKi) have revolutionized therapy and improved the survival of patient subgroups, the overall survival remains rather low, with an average 5-year survival rate of approx. 6% (Doroshow et al., 2019; Ruiz et al., 2019; Pottier et al., 2020).

Genetic profiling, conducted by cancer panel “hot spot” sequencing, whole exome, or next-generation sequencing (NGS), has granted deep insights into the genetic diversity of this disease (Cancer Genome Atlas Research Network, 2012, 2014). Despite this knowledge, the development of therapy resistance is prevailing and limits patient survival (Asao et al., 2019; Lim and Ma, 2019). This is frequently observed in patients undergoing either immune checkpoint or RTKi therapy. Here, either tumors acquire additional mutations and thereby render the applied drug ineffective, or a subclone takes center stage and thrives upon the loss of the previously predominant clone. Furthermore, mutations in additional genes, such as tumor suppressors, can lead to tumor recurrence and treatment resistance.

Lung cancer is subdivided in two major cancer types: non-small cell lung cancer (NSCLC) and small cell lung cancer (SCLC) (Travis et al., 2015). NSCLC in itself is not a genetically simple and homogeneous disease. It represents a rather heterogeneous and highly mutated tumor entity, which is divided by histological features and marker expression into adenocarcinoma (ADC) and squamous cell carcinoma (SCC) (Travis et al., 2015; Mengoli et al., 2018). Several alterations have recently been identified to directly interfere with personalized therapy, such as mutations of *KEAP1* and *STK11/LKB1* (Bonanno et al., 2019; Papillon-Cavanagh et al., 2020). These mutations, either as single or occurring in tandem, negate immunotherapy and negatively affect patient survival. As this is just one example, it highlights the necessity to develop novel surrogate models that allow to swiftly model various somatic mutations found in NSCLC patients, which can be used as therapeutic testbed(s).

Murine models of NSCLC were at the forefront of target validation and drug development for the last decades (Talmadge et al., 2007; Day et al., 2015; Ireson et al., 2019). A steady “workhorse” of these efforts was the NSCLC model established by Tuveson, Jacks, and Berns: the combination of conditional deletion of the whole coding sequence of the tumor suppressor *Trp53* (*Trp53*^*fl/fl*^) in combination with the conditional expression of the oncogenic variant of *KRas* (*KRas^*G12D*^ KRas^*lsl–G12D/wt*^*) (Jackson et al., 2005; DuPage et al., 2009). Loss of *TP53*, concomitant with activating mutations in the small GTPase *KRAS*, is the prime event in NSCLC onset. *TP53* is commonly mutated in 51% of ADC and 82% of SCC samples, respectively, while *KRAS* is predominantly mutant in adenocarcinoma (ADC) but amplified or overexpressed in squamous cell carcinoma (SCC) (Mogi and Kuwano, 2011; Westcott and To, 2013; Wang et al., 2014). In general, KRAS and members of the MAPK pathway are found to be either transcriptionally upregulated or amplified in SCC. Hence, to recapitulate NSCLC in a murine model, mice carrying conditional alleles for Trp53 (*Trp53*^*fl/fl*^) in combination with mutant KRas^*G12D*^ (*Trp53^*fl/fl*^:KRas^*lsl–G12D/wt*^*) represent suitable model systems.

Recognizing the genetic complexity observed in NSCLC and the direct impact mutations have on treatment response highlights the limits of classic mouse models. The introduction of novel genetic alterations requires extensive technical and methodological assets, not available to most research laboratories or facilities. Genetic manipulation by classic murine embryonic stem cell targeting is time and cost intensive; the creation of novel mouse lines (e.g., *via* blastocyst injection) including testing for germline transmission and breeding into the desired genetic background is challenging. Ultimately, combining the newly generated murine mouse line with mice carrying targeted tumor suppressors or oncogenic drivers, such as *KRas*^*lsl–G12D/wt*^ or *Trp53^*fl/fl*^:KRas^*lsl–G12D/wt*^*, generates a tremendous amount of excess animals with undesired genotypes. Furthermore, one is limited to the introduced genetic alterations, such as Cre recombinase-mediated exon deletion or activation of a specific point mutant, such as lsl-*Trp53*^*R172H/wt*^.

With the advent of CRISPR/Cas9, research laboratories are now able to recapitulate various genetic mutations identified in patients without the need of an extensive mouse cohort or advanced facilities (Platt et al., 2014; Sanchez-Rivera et al., 2014). Several laboratories have successfully combined classic NSCLC models with CRISPR-mediated targeting of additional tumor suppressors, demonstrating the potential of CRISPR. However, a direct side-by-side comparison between classic and CRISPR-mediated modeling of the “standard” lung cancer model, *Trp53^*fl/fl*^:KRas^*G12D/wt*^*, has not been done. In this study, we report that tumors induced by either Cre-mediated recombination of conditional alleles (from here onward referred to as *KP*^*GEMM*^) or CRISPR-mediated gene editing (referred to as *KP*^*CRISPR*^) resulted in highly comparable tumors. Similarities were not restricted to histology but extended also to a transcriptional level. Expanding the repertoire of genetic alterations achieved *in vivo* only required standard molecular techniques. Furthermore, tumor induction can even be induced in wild-type mouse strains by the use of a dual viral system. Hence, CRISPR is revolutionizing the way we can conduct tumor research and enables laboratories to address and investigate a plethora of genetic alterations at ease while reducing overall animals required in this particular research field.

## Results

### CRISPR-Cas9 Mediated Editing of *Trp53* and *KRas*^*G12D*^ (*KP*^*CRISPR*^) Induces the Formation of NSCLC That Is Indistinguishable From the Classic GEMM Model *Trp53^*fl/fl*^:KRas^*lsl–G12D/wt*^* (*KP^*GEMM*^*)

**FIGURE 1:**
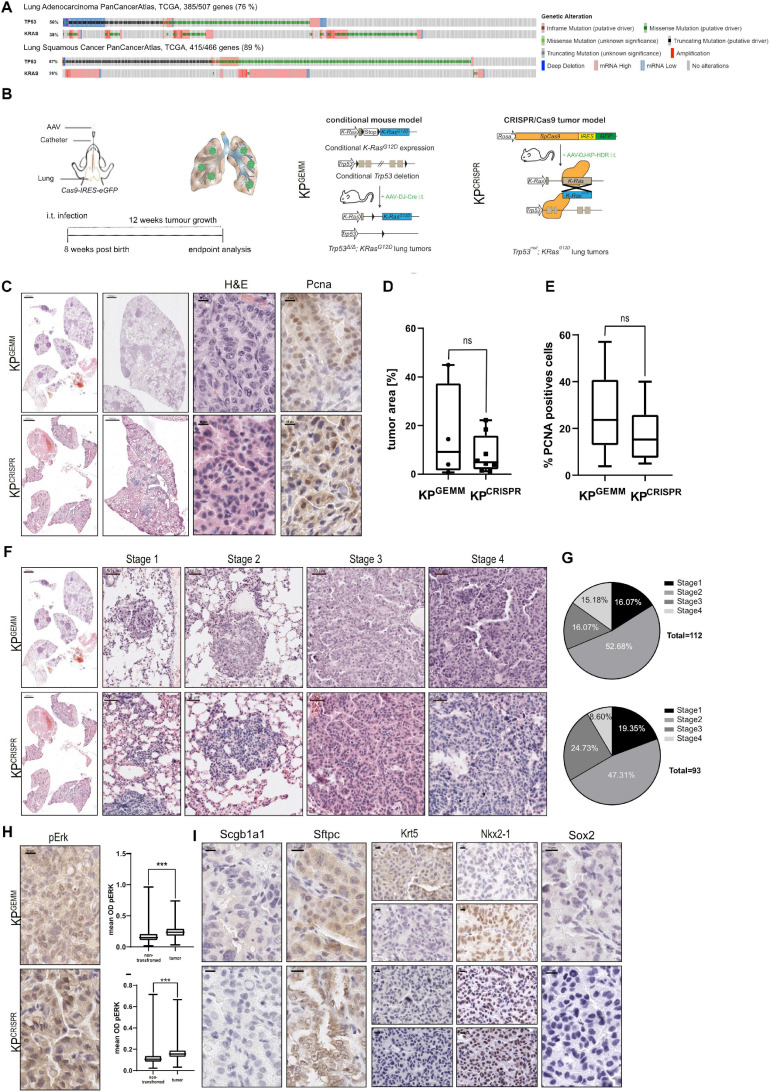
CRISPR/Cas9-mediated editingP of *Trp53* and *KRas*^*G12D*^ (*KP*^*Crispr*^) induces the formation of NSCLC that is indistinguishable from the classic GEMM model *Trp53^*fl/fl*^:KRas^*lsl–G12D/wt*^* (*KP*^*GEMM*^). **(A)** Analysis of occurring alterations in TP53 and KRAS in lung adenocarcinoma (upper panel) and lung squamous cancer (lower panel) (www.cbioportal.org). **(B)** Schematic diagram of the intratracheal tumor induction in *C57BL6/J Trp53^*fl/fl*^:KRas^*lsl–G12D/wt*^* (*KP*^*GEMM*^) *via* AAV-DJ-Cre and CRISPR/Cas9-mediated tumor modeling and targeting of *p53*^Δ^*; KRas^*G12D*^* in *Rosa*^26Sor–CAGG–Cas9–IRES–GFP^ mice (KP^*CRISPR*^). **(C)** Representative H&E images of tumor-bearing animals 12 weeks post intratracheal infection (scale bars 2,000, 1,000, and 10 μm) and representative IHC analysis of PCNA. *KP*^*GEMM*^ *n* = 4; KP^*CRISPR*^ *n* = 8. **(D)** Quantification of % tumor area (normalized to total lung area) in *KP*^*GEMM*^ *n* = 4 and KP^*CRISPR*^ *n* = 8. The *p* value (*p* = 0.3423) was calculated using an unpaired two-tailed *t* test. **(E)** Quantification of % PCNA-positive cells in *KP*^*GEMM*^ and KP^*CRISPR*^ tumors with *n* > 20,000 events, respectively. The *p* value (*p* = 0.2027) was calculated using an unpaired two-tailed *t* test. **(F)** Representative H&E images of tumor stages in *KP*^*GEMM*^ and KP^*CRISPR*^ according to the WHO guidelines from 2015 (scale bars 2,000 and 50 μm). **(G)** Quantification of the tumor stages in *KP*^*GEMM*^ *n* = 112 and KP^*CRISPR*^ *n* = 93. **(H)** Representative IHC staining for pERK in *KP*^*GEMM*^ and KP^*CRISPR*^ and the quantification of the mean optical density (OD) of pERK in adjacent non-transformed tissue and tumors. The *p* value (*p* < 0.0001) was calculated using an unpaired two-tailed *t* test. *KP*^*GEMM*^ *n* > 17,000 and KP^*CRISPR*^ *n* > 18,000. **(I)** Representative IHC staining for Scgb1a1/CC10 and the ADC (SFTPC and NKX2-1) and SCC (Krt5 and Sox2) marker expression of KP^*GEMM*^ and KP^*CRISPR*^ animals. Please also see Supplementary Figure 1.

Loss of TP53, in combination with activating mutations within members of the MAPK pathway, exemplified by alterations in *KRAS*, is the commonly observed driver of human NSCLC (Figure 1A and Supplementary Figure 1A). To assess if CRISPR/Cas9 gene editing can be utilized to recapitulate NSCLC *in vivo*, we compared the classic mouse model *Trp53^*fl/fl*^:KRas^*lsl–G12D/wt*^* (C57BL6/J background, *KP*^*GEMM*^) infected with an adeno-associated virus (AAV) encoding Cre recombinase with *Rosa26*^*Sor–CAG–Cas9–IRES–eGFP*^ mice (C57BL6/J background) infected with an AAV encoding single guide RNA (sgRNA) to target *Trp53* and mutate *KRas* to *KRas*^*G12*^ (*KP*^*CRISPR*^) (Figure 1B and Supplementary Movie 1). Previous studies demonstrated the high degree of fidelity versus off-target mutagenesis by using CRISPR-mediated genome editing *in vivo* (Sanchez-Rivera et al., 2014; Akcakaya et al., 2018). To ensure optimal viral delivery and infection, several AAV capsids were tested (Supplementary Figure 1B). Packaging with the shuffle capsid AAV-DJ yielded the highest infection efficacy. Twelve weeks post intratracheal instillation of virus-containing solutions, *KP*^*CRISPR*^ and *KP*^*GEMM*^ developed tumors and analysis of the oral cavity and trachea of infected mice revealed no *off-target* tumor induction (Figure 1C and Supplementary Figure 2A). Neither tumor burden, as assessed by HE staining, nor tumor cell proliferation, as assessed by % proliferating cell nuclear antigen (PCNA)-positive cells within tumors, revealed significant differences between *KP*^*GEMM*^ and *KP*^*CRISPR*^ (Figures 1D,E). Next, we wondered if tumor grade differs between *KP*^*GEMM*^ and *KP*^*CRISPR*^. Adhering to the established classification system for murine NSCLC revealed no significant differences, as both models presented comparable distributions of all stages, ranging from atypical alveolar hyperplasia (AAH, stage I) to stage IV (Figures 1F,G; Jackson et al., 2001, 2005). Overall animal survival was not affected during the time of analysis by the method used for genetic targeting (12 weeks post i.t., Supplementary Figure 1C). Genetic alterations induced by CRISPR gene editing were confirmed by Sanger sequencing of the respective targeted genomic regions. Successful targeting of *KRas* was confirmed and the integration of the *KRas*^*G12D*^ HDR template detected. For *Trp53*, CRISPR gene editing led to depletions or various lengths, as identified by Sanger sequencing (Supplementary Figure 1D). Oncogenic mutation of *KRas to KRas^*G12D*^* resulted in downstream activation of the MAPK pathway. Phosphorylation of MAPK1/3 (p-Erk1/2) was readily detectable and increased in *KP*^*GEMM*^ and *KP*^*CRISPR*^, compared with adjacent non-tumor tissue (Figure 1H). Analysis of lung lineage-specific marker expression, such as the adenocarcinoma marker thyroid transcription factor-1 (TTF1), the tracheal club cell marker *Scgb1a1* (CC10), or the alveolar type II specific surfactant protein C (SftpC), an additional marker for adenocarcinomas, showed similar expression patterns between *KP*^*GEMM*^ and *KP*^*CRISPR*^ (Figure 1I). One detectable difference was the expression of the basal stem and squamous cell carcinoma marker Krt5. Here, positive tumor cells were only detectable in *KP*^*GEMM*^, while *KP*^*CRISPR*^ showed no positive staining at all (Figure 1I). While Krt5 was positive, Sox2, a marker and driver of SCC, was negative in all samples, while positive cells were detected in basal cells of the trachea, suggesting a co-expression rather than squamous differentiation of tumor cells (Figure 1I and Supplementary Figure 1E). Furthermore, we analyzed *KP*^*GEMM*^ and *KP*^*CRISPR*^ with regard to potential metastasis. Distal organs and prime locations for lung metastasis, such as the liver but also the pancreas and intestine, showed no gross abnormalities nor metastasis in both tumor model systems (Supplementary Figures 2A,B).

These data demonstrate that CRISPR-mediated genome editing of *Trp53* and *KRas* results in tumors which are comparable to the classic GEMM model *Trp53^*fl/fl*^:KRas^*lsl–G12D/wt*^.*

### Abundance of NSCLC-Associated Oncogenic Transcription Factors in *KP*^*GEMM*^ and *KP*^*CRISPR*^ Shows Similarity by Immunohistochemistry

**FIGURE 2:**
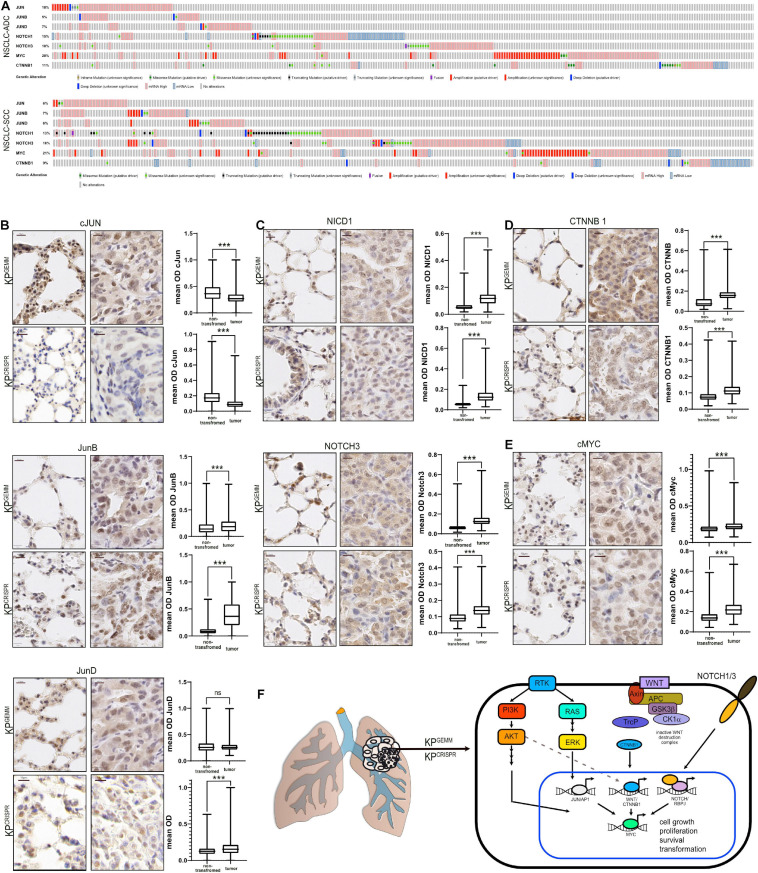
Abundance of NSCLC-associated oncogenic transcription factors in KP^*GEMM*^ and KP^*CRISPR*^ shows similarity by immunohistochemistry. **(A)** Analysis of occurring genetic alterations in lung adenocarcinoma (ADC) and lung squamous cancer (SCC) of associated oncogenes cJun, JUNB, JUND, NOTCH1, NOTCH3, cMYC, and CTNNB1 (www.cbioportal.org). **(B)** Immunohistochemical staining of cJun, JunB, and JunD in KP^*GEMM*^ and KP^*CRISPR*^. Representative non-transformed trachea and tumor areas. Quantification of relative immunohistochemical staining intensity of cJun, JunB, and JunD in non-transformed lung tissue and tumors. Statistical analysis was performed using unpaired *t* test. *P* < 0.0001 (KP^*GEMM*^ JunD *p* < 0.8598). Images were quantified using QuPath (version 0.2.8). Boxplots were generated using GraphPad Prism8. KP^*GEMM*^ *n* > 5,000; KP^*CRISPR*^ *n* > 1,500. *P* values were calculated using two–tailed *t* test statistical analysis. **(C)** Immunohistochemical staining of NICD1 and Notch3 in KP^*GEMM*^ and KP^*CRISPR*^. Representative non-transformed trachea and tumor areas. Quantification of relative immunohistochemical staining intensity of NICD1 and Notch3 in non-transformed lung tissue and tumors. Statistical analysis was performed using unpaired *t* test. *P* < 0.0001. Images were quantified using QuPath (version 0.2.8). Boxplots were generated using GraphPad Prism8. KP^*GEMM*^ *n* > 8,000; KP^*CRISPR*^ *n* > 7,900. *P* values were calculated using two–tailed *t* test statistical analysis. **(D)** Immunohistochemical staining of CTNNB1 in KP^*GEMM*^ and KP^*CRISPR*^. Representative non-transformed trachea and tumor areas. Quantification of relative immunohistochemical staining intensity of CTNNB1 in non-transformed lung tissue and tumors. Statistical analysis was performed using unpaired *t* test. *P* < 0.0001. Images were quantified using QuPath (version 0.2.8). Boxplots were generated using GraphPad Prism8. KP^*GEMM*^ *n* > 7,000; KP^*CRISPR*^ *n* > 5,500. *P* values were calculated using two–tailed *t* test statistical analysis. **(E)** Immunohistochemical staining of cMyc in KP^*GEMM*^ and KP^*CRISPR*^. Representative non-transformed trachea and tumor areas. Quantification of relative immunohistochemical staining intensity of cMyc in non-transformed lung tissue and tumors. Statistical analysis was performed using unpaired *t* test. *P* < 0.0001. Images were quantified using QuPath (version 0.2.8). Boxplots were generated using GraphPad Prism8. KP^*GEMM*^ *n* > 1,900; KP^*CRISPR*^ *n* > 7,000. *P* values were calculated using two–tailed *t* test statistical analysis. **(F)** Schematic of the deregulated oncogenic transcription factors cJun, JunB, JunD, Notch1, Notch3, cMyc, and Ctnnb1 in NSCLC. Please also see Supplementary Figure 2.

Non-small cell lung cancer progression depends on the expression of several oncoproteins, such as the transcription factors cJUN and cMYC (Hartl, 2016; Sanchez-Vega et al., 2018). Furthermore, mutant *KRas*-driven tumors depend on the expression of NOTCH1/3, and the expression of NOTCH pathway members is associated with overall poor survival (Licciulli et al., 2013; Xu et al., 2014). Additionally, WNT signaling is involved in the maintenance of alveolar stem cells and the WNT effector β-catenin is reported to promote oncogenesis of *KRas* mutant NSCLC and is associated with poor survival (Pacheco-Pinedo and Morrisey, 2011; Nakayama et al., 2014). Analysis of publicly available expression data of NSCLC identified the common increase in total abundance for these transcription (co-)factors (Figure 2A). We therefore analyzed the protein abundance of these essential oncoproteins in *KP*^*GEMM*^ and *KP*^*CRISPR*^ and compared the overall abundance relative to non-transformed lung tissue in close proximity to primary tumors (*n* = 10 tumors and *n* > 10,000 cells per analysis, Student’s *t* test, Figures 2B–E). While the expression of the AP-1 transcription factor family member cJun was reduced in tumors compared with adjacent non-tumor tissue, the closely related family members JunB and JunD were significantly upregulated (Figure 2B). This observation was comparable between *KP*^*GEMM*^ and *KP*^*CRISPR*^. Next, we analyzed the total protein abundance of the two NSCLC relevant NOTCH receptors–1 and 3. Here, we performed immunohistochemistry of the cleaved and transcriptionally active form of NOTCH1 (NICD1), as well as total and cleaved NOTCH3 (Figure 2C). In both models, *KP*^*GEMM*^ and *KP*^*CRISPR*^, NICD1 and NOTCH3 were significantly increased in tumors (Figure 2C). β-Catenin was located predominantly in the cytosol in benign lung epithelial cells, with the exception of alveolar type 2 (AT2) stem cells. In tumors, β-catenin/CTNNB1 was predominantly expressed in the nucleus and significantly enriched (Figure 2D). All the aforementioned factors directly or indirectly regulate the expression of the oncoprotein cMYC and thereby converge on pathways regulating proliferation and transformation. We therefore stained for the oncoprotein cMYC (Figures 2E,F). cMYC was significantly upregulated in tumors compared with benign lung tissue, and overall increases were comparable between *KP*^*GEMM*^ and *KP*^*CRISPR*^. We also assessed the protein abundance of the transcription factor ΔNp63, a crucial marker for squamous differentiation (Prieto-Garcia et al., 2020), to re-evaluate the development of SCC in the *KP*^*GEMM*^ and *KP*^*CRISPR*^ models (Figure 1I). This was required since Krt5^+^ tumor cells were detected in *KP*^*GEMM*^ (Figure 1I). In both tumor entities, ΔNp63 was detectable in its respective stem cell niche, with basal stem cells residing in the trachea and bronchus (Wang et al., 2002; Supplementary Figure 2A). However, tumors were negative, indicating that both model systems developed ADC rather than SCC (Supplementary Figures 2A,B).

Tumors developed *via* CRISPR-mediated genome editing of *Trp53* and *KRas* activate the pathologically relevant AP-1, NOTCH, WNT, and MYC pathways, to the same extent as *KP*^*GEMM*^.

### CRISPR-Mediated Targeting of *Trp53* and *KRas* Results in Comparable Adaptations on the Molecular Level of Tumor Cells When Compared With *KP*^*GEMM*^

**FIGURE 3:**
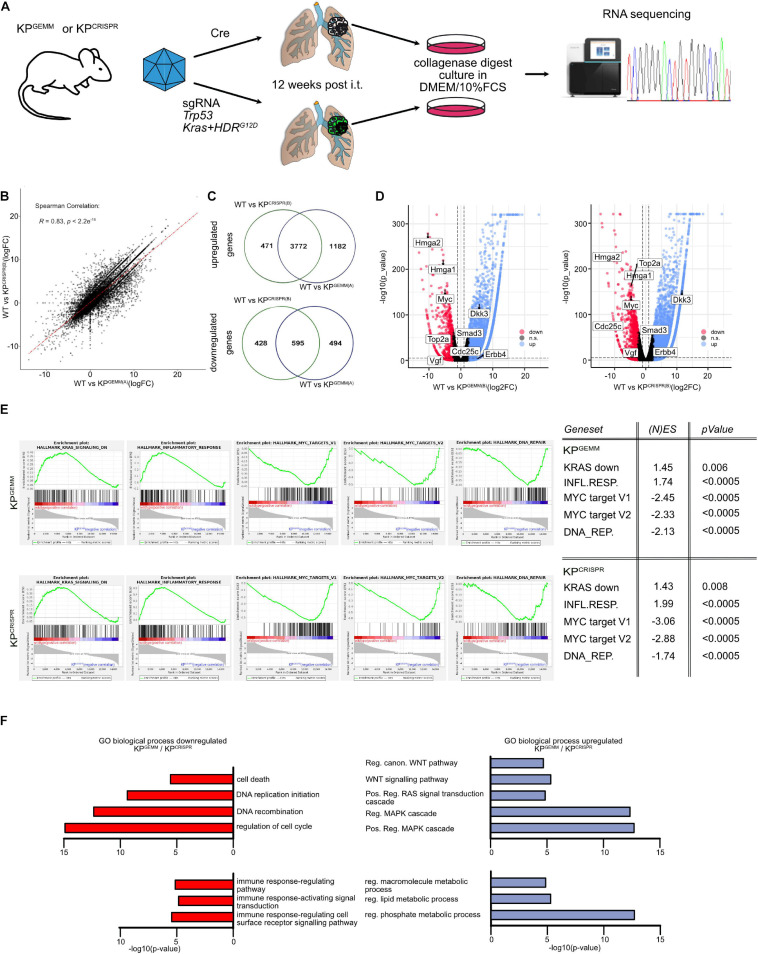
CRISPR-mediated targeting of Trp53 and KRas results in comparable adaptations on the molecular level of tumor cells when compared with KP^*GEMM*^. **(A)** Schematic diagram of the isolation of primary lung tumors from KP^*GEMM*^ and KP^*CRISPR*^, culture, and subsequent RNA-sequencing. **(B)** Correlation plot of differentially regulated genes in WT versus KP^*GEMM(A)*^ and WT versus KP^*CRISPR(A)*^. Spearman correlation of *R* = 0.83, *p* ≤ 2.2e^– 16^. *N* = 3 each. **(C)** Venn diagram of individually and commonly up- and downregulated genes between WT versus KP^*GEMM(A)*^ and WT versus KP^*CRISPR(A)*^. *N* = 3 each (http://bioinformatics.psb. ugent.be/webtools/Venn/). **(D)** Volcano plot of genes up- and downregulated in WT versus KP^*GEMM(B)*^ and KP^*CRISPR(B)*^, respectively. Genes were defined as significant up- and downregulated with log2FC > 2.5. *N* = 3 each. **(E)** Gene set enrichment analyses of KRAS, inflammatory response, MYC target genes, and DNA REPAIR target gene expression in KP^*GEMM*^ and KP^*CRISPR*^. (N)ES, normalized enrichment score and *p* values are depicted in the table. *N* = 3 each. **(F)** GO term analysis over biological processes of commonly down- and upregulated genes in KP^*GEMM(A)*^ and KP^*CRISPR(A)*^, relative to wild-type tissue. *N* = 3 each. Please also see Supplementary Figure 3.

Since *KP*^*GEMM*^ and *KP*^*CRISPR*^-derived primary tumors were indistinguishable on a microscopic scale and are driven by the same oncogenic pathways, we wondered if the similarities extend to the molecular level. To address this question, we isolated primary tumors 12 weeks post induction from *KP*^*GEMM*^ and *KP*^*CRISPR*^ and generated isogenic cell lines and subjected these to whole transcriptome analysis by RNA-sequencing (Figure 3A). As a control, we used wild-type lung tissue samples. Comparison of the transcriptional profile of two individual *KP*^*GEMM*^ and *KP*^*CRISPR*^ cells by RNA-sequencing revealed an overall similar transcriptomic profile (Spearman correlation *R* = 0.83, *p* < 2.2^*e–16*^, Figure 3B and Supplementary Figure 3A). In response to oncogenic transformation, *KP*^*GEMM*^ and *KP*^*CRISPR*^ tumor cells commonly downregulated ∼3,600 and upregulated ∼700 genes (logFC -2.5/+2.5 cutoff, Figure 3C and Supplementary Figure 3B). Among the commonly upregulated genes were the Hmga1 and 2, which encode for chromatin-associated proteins regulating transcription and being involved in metastasis; the oncogene cMYC; topoisomerase 2a; VGF; Smad3; and Cdc25c, a regulator of cell division. On the other hand, Erbb4 and Dkk3 were commonly downregulated factors (Figure 3D).

To gain insights into the biological processes that are commonly up- or downregulated, we performed Gene Set Enrichment Analysis and GO term pathway analysis (Figures 3E,F). Here, we identified that gene sets associated with negative regulation upon oncogenic KRAS signaling as well inflammatory responses were downregulated in *KP*^*GEMM*^ and *KP*^*CRISPR*^ tumor, while tumor cells upregulated MYC target gene signatures and DNA repair pathways (Figure 3E). Not only were these factors deregulated in the murine cell lines but also showed a comparable expression in human NSCLC tumors, as seen by the analysis of publicly available datasets (Supplementary Figure 3D).

GO term pathway analysis identified pathways associated with cell death and cell cycle control that were commonly downregulated in *KP*^*GEMM*^ and *KP*^*CRISPR*^ tumor cells (Figure 3E). Furthermore, we found that pathways associated with immune responses were also toned down (Figure 3E). This is in line with recent publications demonstrating that mutations in the *KRas* oncogene contribute to immune-evasive phenotypes (van Maldegem and Downward, 2020). Analyzing pathways upregulated in tumor cells, we could identify that *KP*^*GEMM*^ and *KP*^*CRISPR*^ are driven by aberrant MAPK signaling and oncogenic Ras, as these pathways were significantly upregulated (Figure 3E). Not only did we identify increased nuclear Ctnnb1 abundance by histopathology, but we also observed an increase in WNT pathway activation by GO term analysis (Figure 3E). To accommodate increased proliferation and oncogenic transformation, tumor cells also upregulated metabolic pathways, such macromolecule synthesis and lipid and phosphate metabolism processes (Figure 3E).

It is noteworthy, despite all the similarities between *KP*^*GEMM*^ versus *KP*^*CRISP*^ when directly compared (*KP*^*GEMM*^ vs. *KP*^*CRISP*^, Spearman correlation *R* = 0.93, Supplementary Figure 3E), that several genes are differentially regulated between *KP*^*GEMM*^ and *KP*^*CRISPR*^ (*KP*^*CRISPR*^ up *n* = 1,612, *KP*^*GEMM*^ up *n* = 918, Supplementary Figure 3F). As seen by Sanger sequencing of primary tumors, *KP*^*CRISPR*^ predominantly harbored homozygous *KRas*^*G12D*^; in contrast, *KP*^*GEMM*^ only carried one mutant allele (Supplementary Figure 1E; Chiosea et al., 2011). While gene expression of major key players in NSCLC, such as Keap1, Stk11/Lkb1, Kras, cJun, cMyc, Notch1, Notch3, or Nkx2-1, was not altered, *KP*^*CRISPR*^ showed an increase in the expression of genes involved in Wnt signaling, while *KP*^*GEMM*^ upregulated the Egfr signaling pathway (Supplementary Figures 3F,G).

Not only do *KP*^*GEMM*^ and *KP*^*CRISPR*^ show a high degree of similarity on a macroscopic scale, these data also demonstrate that classic and CRISPR-mediated targeting of *Trp53* and *KRas* results in similar alterations on a molecular level. Furthermore, our data clearly highlighted that CRISPR-mediated genome editing is a suitable replacement of classic mouse models of NSCLC relying on the targeting of *Trp53* and *KRas*.

### Rapid Modeling of Common Loss-of-Function Mutations Identified in NSCLC Patients by CRISPR Genome Editing

**FIGURE 4:**
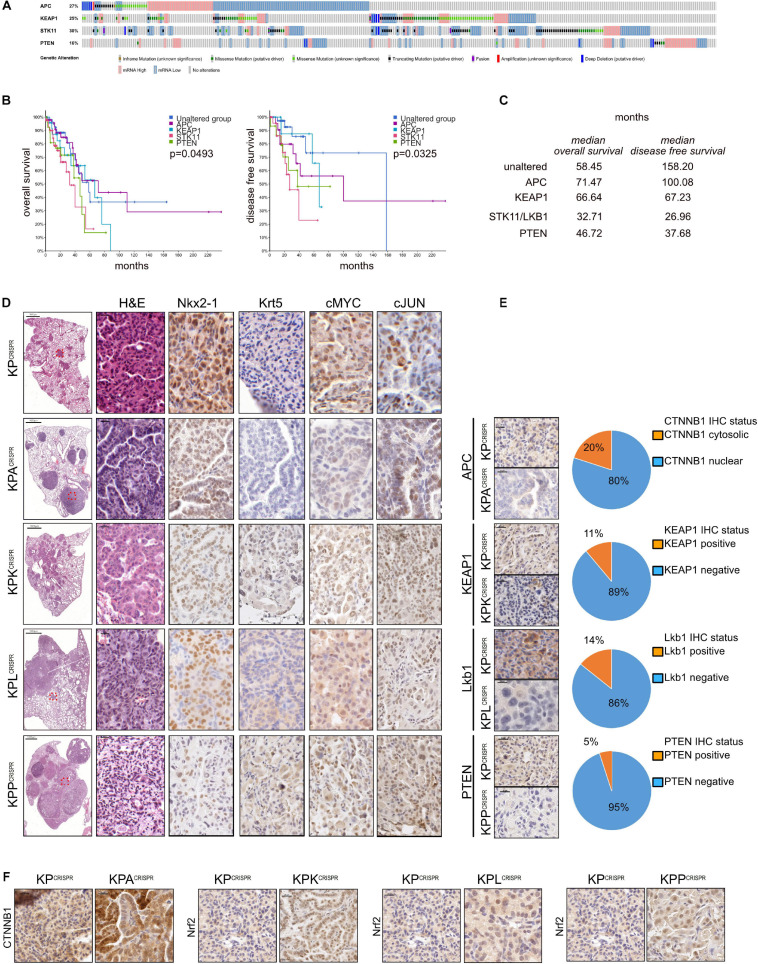
Rapid modeling of common loss-of-function mutations identified in NSCLC patients by CRISPR genome editing. **(A)** Analysis of occurring alterations in APC, KEAP1, STK11/LKB1, and PTEN in human NSCLC-ADC (www.cbioportal.org). **(B)** Kaplan–Meier plot of overall and disease-free survival in NSCLC-ADC patients. Shown are the months’ survival. Blue = unaltered group; purple = APC altered; blue = KEAP1 altered; orange = STK11 altered; green = PTEN altered. *P* < 0.005. **(C)** Median overall and disease-free survival of patients with the indicated alterations, relative to patients with no mutations in APC, KEAP1, STK11, or PTEN. **(D)** Representative H&E and immunohistochemical staining against ADC (NKX2-1) and SCC (Krt5) marker expression and the oncogenes cMyc and cJun of mice infected with AAV encoding sgRNA targeting KP, KP-Apc, KP-Keap1, KP-Lkb1, or KP-Pten. Scale bar 20 μm. **(E)** Representative immunohistochemical staining against encoding sgRNA targets Apc, Keap1, Lkb1, and Pten in KP, KP-Apc, KP-Keap1, KP-Lkb1, or KP-Pten. Scale bar 20 μm. Quantification of CTNNB1 nuclear to cytosolic ratio in KP versus KPA tumors. Quantification of Keap1-positive to negative tumors in mice infected with AAV encoding sgRNA targeting Keap1. Quantification of Lkb1/Stk11-positive to negative tumors in mice infected with AAV encoding sgRNA targeting Lkb1/Stk11. Quantification of Pten-positive to negative tumors in mice infected with AAV encoding sgRNA targeting Pten. **(F)** Representative immunohistochemical staining against Ctnnb1 in KP^*CRISPR*^ and KPA^*CRISPR*^, Nrf2 in KP^*CRISPR*^ and KPK^*CRISPR*^, Nrf2 in KP^*CRISPR*^ and KPL^*CRISPR*^, and Nrf2 in KP^*CRISPR*^ and KPP^*CRISPR*^. Please also see Supplementary Figure 4.

Non-small cell lung cancer is one of the most mutated solid tumors known (Cancer Genome Atlas Research Network, 2012, 2014). Analysis of publicly available datasets demonstrates the frequent loss of various tumor suppressors. Among the most commonly mutated are E3 ligases, such as *APC* or *KEAP1*, key enzymes of the ubiquitin system, as well negative regulators of (stem) cell growth and transformation, such as *STK11/LKB1* and *PTEN* (altered in 403/503 ADC (80%) and 437/477 SCC (94 (Figure 4A and Supplementary Figure 4A). Furthermore, mutations/alterations of these tumor suppressors negatively correlated with patient survival (Figure 4B). While loss of STK11 and PTEN significantly reduced overall survival (32.71 or 46.72 vs. 58.45 months, respectively), mutations in any of the four tumor suppressors negatively affected disease-free and progression-free survival (Figure 4C and Supplementary Figures 4B,C). To assess if CRISPR can be used to swiftly model these particular genetic alterations, we introduced an additional sgRNA cassette, comprising the murine U6 promoter, the guide RNA and trcRNA hybrid, into the *KP*^*CRISPR*^ AAV backbone (Supplementary Figure 4D). Twelve weeks post viral application, mice were analyzed by immunohistopathology. Loss of the aforementioned tumor suppressors, *APC*, *KEAP1*, *STK11/LKB1*, and *PTEN*, significantly enhanced tumor burden and increased tumor cell proliferation when compared with *KP*^*CRISPR*^ (Figure 4D and Supplementary Figure 4E). While mutations of *Apc* and *Keap1* led to the development of ADC, targeting of *Stk11/Lkb1* and *Pten* also led to the development of SCC tumors (Krt5^+^, P63^+^, TTF1^–^) (Figure 4D; Prieto-Garcia et al., 2020). Loss of these factors also resulted in an increase in protein abundance of the oncogenes cMYC and cJun (Figure 4D). CRISPR-mediated genome editing and tumor suppressor deletion were confirmed by immunohistochemistry. Apc, Keap1, Lkb1, and Pten were not detectable in the majority of developing tumors at endpoint, confirming successful genomic targeting of the tumor suppressors on the protein level (targeting efficacy as assessed by IHC: Apc: 80%; Keap1: 89%; Lkb1: 86%; Pten: 95%, Figure 4E). Targeting was further confirmed by IHC staining against known downstream target proteins. Loss of *Apc* resulted in the nuclear accumulation of β-catenin, while deletion of *Keap1* led to the accumulation of its bona fide substrate, Nrf2/Nfe2l2 (Figure 4F). Targeting of *Stk11/Lkb1* significantly upregulated tumor burden and tumor cell proliferation. These tumors also showed elevated protein abundance of Nrf2/Nfe2l2 (Figure 4E). Loss of Stk11/Lkbn1 impacts tumor cell metabolism and increases ROS production, while elevated abundance of Nrf2 establishes ROS homeostasis, enabling tumor cells to grow under stress conditions (Ma, 2013; Faubert et al., 2014; Kaufman et al., 2014; Galan-Cobo et al., 2019). Lastly, loss of Pten strongly synergized with mutations in *Trp53* and *KRas*. Tumors devoid of Pten accumulated Nrf2/Nfe2l2 as well, which is in line with published data (Figure 4E; Rojo et al., 2014; Best et al., 2018).

CRISPR-mediated genome editing *in vivo* allows swift modeling of complex genetic alterations that are clinically relevant and occur in human NSCLC patients. It can therefore serve as a relevant surrogate model and potentially be used for therapy development.

### Dual Viral Approaches Enhance the Applicability of CRISPR for Translational Research, Irrespective of the Mouse Strain

**FIGURE 5:**
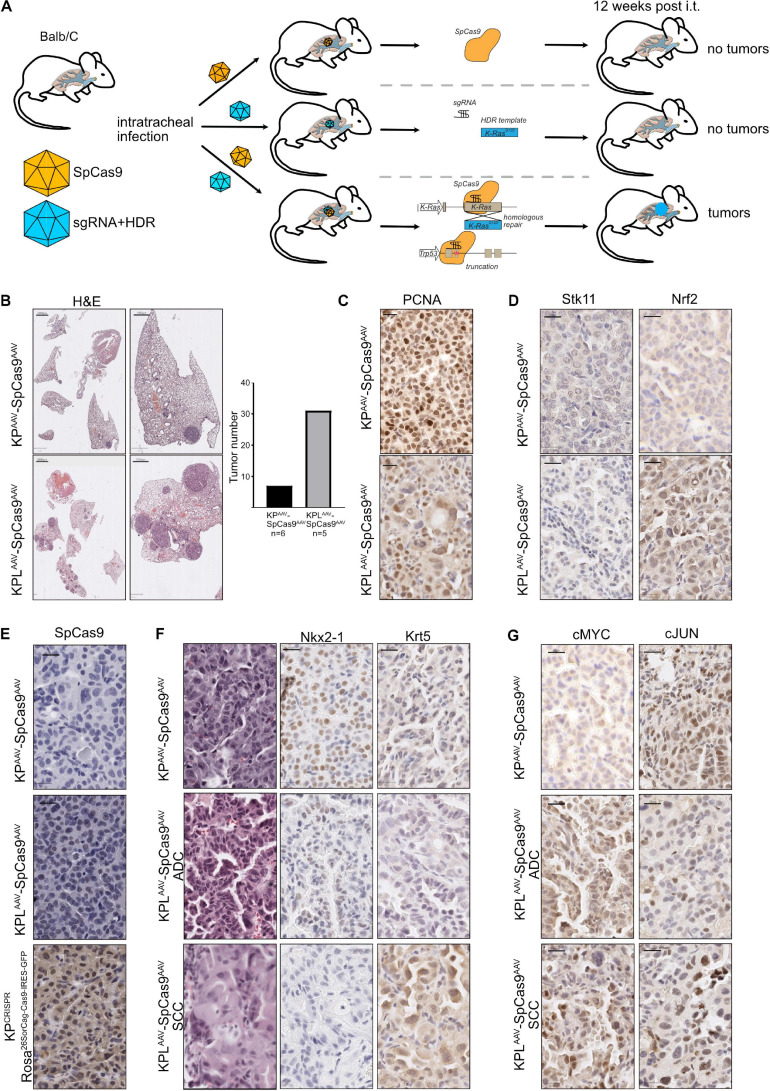
Dual viral approaches enhance the applicability of CRISPR for translational research, irrespective of the mouse strain. **(A)** Schematic diagram of the dual AAV approach of intratracheal infection of mice to induce spCAS9-mediated lung tumor formation. **(B)** Representative H&E images of tumor-bearing animals 12 weeks post intratracheal infection (scale bars 2,000 and 1,000 μm). Quantification of absolute tumor numbers in all KP or KPL animals co-infected with an AAV-spCas9. *N* = 6. **(C)** Representative IHC images of PCNA in KP^*AAV*^SpCas9^*AAV*^ and KPL^*AAV*^SpCas9^*AAV*^ (scale bar 20 μm). **(D)** Representative IHC images of Lkb1/Stk11 and Nrf2 in KP^*AAV*^SpCas9^*AAV*^ and KPL^*AAV*^SpCas9^*AAV*^ (scale bar 20 μm). **(E)** Representative IHC images of spCas9 in KP^*AAV*^SpCas9^*AAV*^ and KPL^*AAV*^SpCas9^*AAV*^ and KP^*CRISPR*^ (Rosa^26SorCag–Cas9–IRES–GFP^) (scale bar 20 μm). **(F)** Representative IHC staining for Nkx2-1 and Krt5 in KP^*AAV*^SpCas9^*AAV*^ and KPL^*AAV*^SpCas9^*AAV*^ (scale bar 20 μm). **(G)** Representative IHC staining for cMyc and cJun in KP^*AAV*^SpCas9^*AAV*^ and KPL^*AAV*^SpCas9^*AAV*^ (scale bar 20 μm).

To test if CRISPR-mediated gene editing can be applied *in vivo* without the need of a constitutive Cas9-expressing mouse strain, we used a dual AAV virus infection protocol (Figure 5A). Here, one AAV virion encoded the SpCas9 enzyme under the control of a short EFS promoter, while the second virion encoded the components necessary to target *Trp53* and *Stk11/Lkb1* and to oncogenic mutate *KRas* to *KRas*^*G12D*^, *KP*^*CRISPR*^, or *KPL*^*CRISPR*^, respectively. Balb/C mice were infected *via* the tracheal route with a mix of 2 ^∗^ 10^8^ GC (genomic copies) of each virus in a total volume of 60 μl and tumor burden 12 weeks post infection was analyzed. Histopathological analysis of the dual viral-infected animals to single virus *KP*^*CRISPR*^ revealed that, while total tumor numbers were significantly reduced (*KP*^*CRISPR–double*^ *n* = *x* to *KP*^*CRISPR*^ *n* = *y*), no gross morphological differences with regard to tumor grade were observed as both models displayed the development of stage I to stage IV tumors (Supplementary Figure 5A). The expression of lineage-specific markers, such as Nkx2-1/TTF-1 and Krt5, was comparable and tumors displayed activation of the Erk pathway, as seen by IHC staining against phosphor-Erk1/2 (Supplementary Figures 5B,C). Overall expression and abundance of oncoproteins was comparable with the *KP*^*CRISPR*^ model system (Figure 2 and Supplementary Figure 5D). Loss of *Lkb1*, in combination with deletion of *Trp53* and mutation of *KRas*, resulted in increased tumor incidence and larger tumors, when compared with *KP* targeting alone (Figure 5B). Proliferation was increased in KPL compared with KP and was comparable with the aforementioned KPL model system (Figures 5C and 4E). Successful targeting of Stk11 was confirmed by immunohistochemistry (Figure 5D). While constitutive SpCas9 enzyme expressing C57Bl6/J mice showed positive immunoreactivity with a SpCas9-specific antibody, the dual AAV-infected animals were devoid of staining (Figure 5E). Targeting of Stk11 also led to the establishment of KRT5^+^/TTF1^–^ tumors, indicating that loss of this tumor suppressor leads to the simultaneous development of NSCLC-SCC and NSCLC-ADC (Figure 5F). This is in line with a previous observation we made in a constitutive CRISPR-driven mouse model (Prieto-Garcia et al., 2020). cMYC protein levels were elevated in tumors and cMyc and cJun total protein abundance was comparable to *KP*^*GEMM*^ and *KP*^*CRISPR*^ animals (Figures 4E, 5G).

Thus, our data shows that CRISPR gene editing enables tumor onset irrespective of the mouse strain and does not require the constitutive expression of the enzyme Cas9.

## Discussion

### CRISPR-Mediated Genome Editing of Oncogenes and Tumor Suppressors Establishes Similar Histologic and Molecular Features of Human NSCLC

Genetic modifications or deletions of tumor suppressors, such as *Trp53*, in combination with activating mutations in proto-oncogenes, such as *KRas*, are a prerequisite to facilitate a tumor-initiating event (Best et al., 2019). While for the last two decades in mouse models, loss of tumor suppressors was initiated by either germ line deletion or conditional loss (mediated by the activity of a recombinase such as Cre), we wondered if CRISPR-mediated genome editing is capable of facilitating similar effects.

Comparing primary tumors, established by either Cre recombinase-mediated genetic loss of *Trp53* and activation of a dominant mutant *KRas*^*G12D*^ allele (*KP*^*GEMM*^) versus CRISPR-mediated genomic targeting and mutation (*KP*^*CRISPR*^), we could not detect gross differences on a microscopic level. Mice had a comparable tumor burden and established tumors had a comparable level of proliferative cancer cells. Overall expression of lung and tumor lineage markers was indistinguishable, demonstrating that the genetic alterations inflicted, and not the method used, define the histopathologic features or primary tumors. On the other hand, for primary lung tumors of various grades, ranging from I to IV, we did not detect any metastasis within the analyzed time frame of 12 weeks post tumor induction. This observation is in line with previous reports, as the onset of metastasis, which occurs in *Trp53^*R173H/wt*^:KRas^*lsl–G12D/wt*^* conditional mice at a late time point exceeding 12 weeks (Zheng et al., 2007). It will be interesting for future work to extend the time frame to allow and investigate if *KP*^*CRISPR*^ animals do form metastasis.

It is noteworthy, however, that, in contrast to *KP*^*CRISPR*^, *KP*^*GEMM*^ also developed with low-frequency primary tumors expressing the SCC markers Krt5 while being negative for the pathological relevant markers ΔNp63 and Sox2. It could be possible that these tumor cells are on the *trans-*differentiation to SCC. We previously reported the expression of SCC markers in this mouse model, and others have reported that *trans-*differentiation can occur in KRas^*G12D*^-driven ADC to SCC (Han et al., 2014; Prieto-Garcia et al., 2020). Despite the extensive use of the *KP*^*GEMM*^ model in the past, it was reported to exclusively only give rise to ADC (Jackson et al., 2001). In contrast to other publications, where adenoviruses or lentiviruses were utilized to transmit genomic information, we used AAV virions for Cre recombinase delivery. As loss-of-function mutations in *Trp53*, concomitant with aberrant MAPK pathway signaling, are common features found in ADC and SCC, we assume that the viral tropism of the AAV shuffle capsid DJ is responsible for this observation.

Similarities between *KP*^*CRISPR*^ and *KP*^*GEMM*^ are not limited to the expression of histopathologic markers, but extend to the transcriptional programs identified in tumor cells. Irrespective of the targeting strategy, tumor cell programs adapted to transformation by downregulating cell death programs as well the immune response signatures. As the analyzed tumors were driven by oncogenic mutations in *KRas*, several key pathways were found to be upregulated, including MAPK, RAS, and WNT. To accommodate enhanced proliferation, tumors also readjusted various metabolic pathways. One significant difference we identified between the two NSCLC models is that targeting of *KRas* by CRISPR led to a homozygous recombination event, resulting in *KRas*^*G12D/G12D*^. This is in contrast to the genetically predefined heterozygous expression of the oncogenic *KRas*^*G12D/wt*^ in the *KP*^*GEMM*^ models. As homozygous breeding of the conditional mouse model is not feasible due to embryonic lethality observed in biallelic *KRas*^*lsl–G12D/lsl–G12D*^ embryos, CRISPR enables us to model and analyze the consequences of homozygous mutations in KRas, as NSCLC patients can present both genetic modifications (Chiosea et al., 2011; Ferrer et al., 2018).

### Simple Modifications of the CRISPR Toolbox Allows for the Expansion of the Mutational Repertoire of NSCLC

To expand the repertoire of targeted alleles by CRISPR, one only requires to introduce additional sgRNA cassettes with a high *on-target*, low *off-target* rates. As we reported in our study, the implementation of CRISPR helps to speed up the time required from target identification to *in vivo* validation tremendously.

Cloning into available viral vectors encoding for lentivirus or AAV, followed by subsequent virus production, can be completed within one and a half weeks and requires minimal financial investment. This is an improvement in flexibility not seen before which will significantly change the way we will conduct our research in the future. With the development of novel Cas9 systems, the *off-target* rate can be significantly reduced (Naeem et al., 2020).

One has to consider, however, that the implementation of CRISPR for target gene modification will result in individual and heterogeneous genetic events. Here, the classic conditional targeting of genetic loci will produce genetically identical recombination events. Depending on the scientific question one will address, the classic mouse model can be advantageous.

Our data demonstrated that for gene deletions induced by CRISPR targeting of the tumor suppressor *Trp53*, the overall molecular changes were comparable among individually tested cell lines, but not identical. Hence, primary tumor cell lines generated by CRISPR should be treated as individual tumor lines and not as clones.

A previous work, pioneered by the Tyler Jacks Laboratory, successfully combined classic conditional *Trp53^*fl/fl*^:KRas^*lsl–G12D/wt*^* animals with lentiviral delivery of the CRISPR enzyme Cas9, guide RNA targeting similar tumor suppressor as presented in our study (*Apc* and *Pten*), and the Cre recombinase to induce loss of *Trp53* and activate the expression of the oncogenic *KRas*^*G12D*^ allele (Sanchez-Rivera et al., 2014). This strategy enabled the rapid expansion of already existing *in vivo* models and also enabled the long-term expression of target genes or encoded additional genetic information, such as shRNAs (Sanchez-Rivera et al., 2014; Xue et al., 2014; Ng et al., 2020). Lentiviruses, in comparison to AAV, have a significantly higher total packaging capacity (10 vs. 4.8 kb); however, they are stably integrated and advertently can result in the disruption of additional genetic loci, including tumor suppressors, while AAV reside as episomal circles in infected cells and integrate with very low frequency (<0.05%) (Inagaki et al., 2008; Yang et al., 2008). A second strategy is the use of adenoviruses, encoding sgRNA and Cre recombinase. Here, the AdEasy I and II recombinant viral production system has enabled researchers for several years to produce high-titer viruses (Luo et al., 2007). Similar to AAV, AdV also reside as episomes in infected cells, but are rapidly cleared. While packaging capacity is higher than in AAV (AdEasy I 7.5 kb, AdEasy II 12 kb), recombination of the pShuttle vector with the AdEasy I backbone can prove difficult and research labs require biosafety level II tissue cultures to produce and propagate adenoviruses and lentivirus (Collins et al., 2017). AAV, on the other hand, are nonpathogenic and high-titer viruses can be produced with ease in biosafety level I tissue cultures.

### CRISPR Meets 3R: Direct Implementation of Reduce and Refine by Switching From GEMM to CRISPR for NSCLC Research

To investigate the putative tumor-suppressive or oncogenic role of a protein in NSCLC, laboratories would have to generate and establish a novel mouse line, which is a time-consuming endeavor and limited by the availability of the required facilities. Alternatively, one would purchase an available genetically engineered mouse model from commercial suppliers harboring the (available) conditional alleles. Both methods require the combination of the pre-existing mouse strains with the newly acquired lines, necessitating at least two to three generations of breeding until the desired genotype becomes available. Under optimal conditions, this process alone can take up to half a year prior to starting the experiment.

**FIGURE 6:**
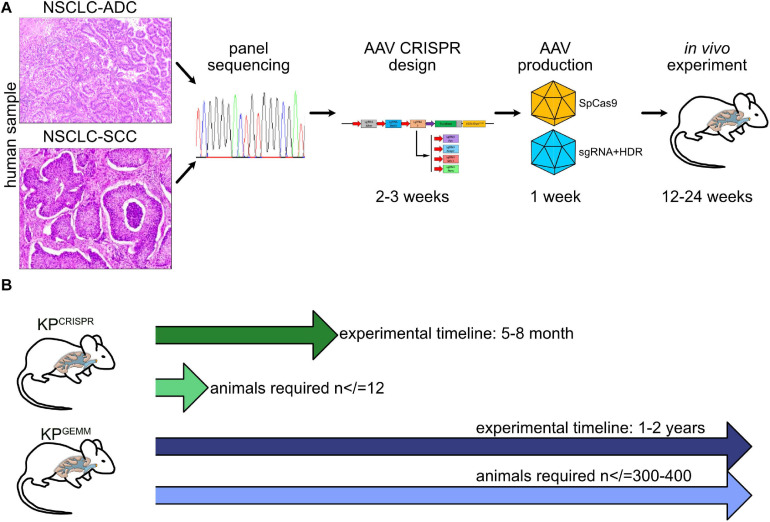
Schematic model of CRISPR NSCLC models. **(A)** Workflow from “bed” to *in vivo* tumor model by CRISPR. **(B)** Comparison of time frame and animals used between KP^*GEMM*^ and KP^*CRISPR*^.

Introducing CRISPR gene targeting, at least for NSCLC, helps to implement the 3R guidelines for *in vivo* research: it significantly reduces the overall amount of generated animals (*Reduce*) and, with applied biostatistics, reduces animals used for experiments even further (*Refine*). Our strategies reported in this study can also be applied to organoid model systems and could therefore also help to partially *Replace in vivo* tumor models (Figure 6).

CRISPR has revolutionized the way we conduct our research. It is here to stay and will reshape cancer research for the foreseeable future.

## Materials and Methods

### Tissue Culture and Reagents

The HEK-293T cell lines were obtained from Agilent. HEK-293T cells were cultured in DMEM (Gibco) supplemented with 10% fetal bovine serum (FCS)/1% Pen-Strep. Cell lines were authenticated by STR profiling. Cells were routinely tested for mycoplasma *via* PCR.

### DNA Transfection and Infection

DNA transfection was performed by adding a mix of 2.5 μg plasmid DNA, 200 μl serum-free medium, and 5 μl PEI to the cells seeded in a six-well plate (60% confluence). After 6 h incubation at 37°C, the medium was changed to full supplemented medium, and finally, cells were collected after 48 h for experimental purposes. For viral infection, AAVs or lentiviruses (MOI = 10) were added to the medium in the presence of polybrene (5 μg/ml) and incubated at 37°C for 4 days. The selection of infected cells was performed with 2.5 μg/ml puromycin for 72 h, 250 μg/ml neomycin for 2 weeks, or FACS-sorting RFP/GFP-positive cells (FACS Canto II BD).

### Primary Murine Lung Cancer Cell Lines

Primary lung cancer cell lines were obtained from 12-week-old mice as previously described (Prieto-Garcia et al., 2020). At the endpoint of the experiment, mice were sacrificed and lung tumors isolated. Tissue was digested in collagenase I (100 U/ml in PBS for 30 min at 37°C), and after stopping the reaction with FCS, the mixture was centrifuged and resuspended in DMEM (Gibco) supplemented with 10% fetal bovine serum (FCS) and 1% Pen-Strep. Fibroblasts were counter-selected by selective trypsinization and homogeneous cell clusters were clonally expanded.

### Immunohistochemistry

Primary lung cancer samples were obtained from 12-week-old mice as previously described (Prieto-Garcia et al., 2020) and embedded in paraffin and sectioned at 4 μm using a microtome (Leica). Before staining, slides were deparaffinized and rehydrated using the following protocol: 3 × 5 min in Histo-Clear^®^, 2 × 3 min in EtOH (100%), 2 × 3 min in EtOH (95%), 2 × 3 min in EtOH (70%), 3 min in EtOH (50%), and 3 min in H_2_O. Slides were stained with hematoxylin and eosin. For IHC, primary antibodies were incubated at 4°C or for 3 h at 37°C, followed by subsequent incubation with the secondary antibody for 1 h at room temperature. After antibody exposure, slides were washed twice with TBS and stained with the DAB staining solution. The stained samples were mounted with Cytoseal^TM^ 60, and IHC images were recorded using either Pannoramic DESK scanner DW II or Roche Ventana DP 200 slide scanner and analyzed with the CaseViewer software (3DHISTECH) and QuPath^1^ ^,2^ as indicated at the website^3^.

### sgRNA Design

sgRNAs were designed using the CRISPRtool^4^.

### AAV Production and Purification

Viruses were synthetized in HEK-293T cells. For AAV production, cells were co-transfected with the plasmid of interest (10 μg), pHelper (15 μg), and pAAV-DJ (10 μg) using PEI (70 μg/15 cm dish). AAV virus isolation from transfected cells was performed as previously described (Prieto-Garcia et al., 2020).

In detail, for the production of AAVs, *5* ^*106^ HEK-293T cells were seeded in 15 cm cell culture dishes and cultivated for 24 h or until a confluence of ∼60–70% was achieved. In order to gain a high titer, three to four plates for one virus were used, respectively, and pooled during harvest.

Cells were transfected with the pRepCap (pRC), the *cis-*plasmid (pAAV), and the pAdDeltaF6 (Table 1) in a 1:1:2 molar ratio. Therefore, the DNA was mixed in 2 ml DMEM (w/o FCS), and polyethylenimine (PEI) (DNA:PEI ratio of 1:2, e.g., 45 μg DNA = 90 μg PEI) was added. The mixture was incubated 15 min at room temperature and added dropwise to the plates.

**TABLE 1:** DNA amount for AAV plasmids.

**Plasmid**	**μ g**	**μ g/ml**	**fmol/μ l**	**Molar ratio**
pRC	10	0.66	0.14	1
pAAV	10	0.66	0.15	1
pAdDeltaF6	25	1.66	0.30	2

To harvest the AAV, cells and the supernatant were collected after 96 h and transferred into a 50-ml conical tube. At first, NaCl was added (f.c. 0.5 M) and slowly mixed for 1 h at 4°C. Next, chloroform was added (f.c. 10%) and slowly mixed for 30 min at 4°C. Eventually, the suspension was centrifuged at 2,000 × *g* for 30 min at 4°C. The water phase was transferred into a new conical tube and PEG 8,000 was added (f.c. 10%) and mixed well. The AAV can be precipitated for 90 min or overnight at 4°C.

After the centrifugation at 2,000 × *g* for 20 min at 4°C, the pellet was dissolved in PBS Mg^2+^/Ca^2+^ or AAV resuspension buffer (PBS + 0.001% Pluronic F68 + 200 mM NaCl) (∼100 μl/15 cm dish used), and protease inhibitor and DNase/RNase were added (f.c. 1 ×). Then, it was incubated for 2 h at 37°C, added with chloroform (1:1 ratio), and centrifuged at 12,000 × *g* for 5 min at 4°C. The chloroform step was repeated and the water phase was collected. Finally, the product was titrated or stored at −80°C.

### *In vivo* Experiments and Histology

All *in vivo* experiments were approved by the Regierung Unterfranken and the ethics committee under license numbers 2532-2-362, 2532-2-367, 2532-2-374, and 2532-2-1003. The mouse strains used for this publication are listed. All animals are housed in standard cages in pathogen−free facilities on a 12−h light/dark cycle with *ad libitum* access to food and water. FELASA2014 guidelines were followed for animal maintenance.

Adult mice were anesthetized with isoflurane and intratracheally intubated with 60 μl AAV virus (3 × 10^7^ PFU) as previously described (Prieto-Garcia et al., 2020). Viruses were quantified using the AAV Titration by qPCR protocol from Addgene (Aurnhammer et al., 2012). Animals were sacrificed by cervical dislocation and lungs were fixed using 5% NBF.

### RNA-Sequencing

RNA concentration was determined with a NanoDrop spectrophotometer and analyzed on a Fragment Analyzer (Advanced Analytical) for RNA quality; 400 ng of RNA was then used and enriched for poly(A) mRNA using the NEBNext Poly(A) mRNA Magnetic Isolation Module (#E7490L) according to the manufacturer’s instructions. The fragmentation time was 15 min at 94°C for every sample [RNA quality number (RQN) > 8], except for the three wild-type replicates that showed an RQN between 4 and 7. For those samples, the fragmentation time was reduced to 7.5 min. After poly(A) mRNA enrichment and fragmentation, the NEBNext^®^ Ultra^TM^ II Directional RNA Library Prep Kit for Illumina^®^ (#E7760L) was used for library preparation according to the manufacturer’s instructions with 9 cycles of PCR amplification.

RNA-sequencing was performed with Illumina NextSeq 500 as described previously (Buchel et al., 2017).

### Quantification and Statistical Analysis

#### RNA-Sequencing Analysis

Base calling was performed using Illumina’s BaseSpace platform and overall sequencing quality was analyzed using the multiQC script. Reads were aligned to the murine genome (mm10) using Bowtie2 v2.4.1 (Langdon, 2015), and samples were normalized to the number of mapped reads in the smallest sample. For differential gene expression analysis, reads per gene were counted with the “featureCounts” function from the R (version 4.0.3) package “GenomicFeatures” (version 1.40.1), and none or weakly expressed genes were removed (mean read count over all samples <6). Differentially expressed genes were called using edgeR (Robinson et al., 2010) and the resulting *p* values were corrected for multiple testing by false discovery rate (FDR) calculations by the Benjamini–Hochberg method. GSEA analyses (Subramanian et al., 2007) were done with Signal2Noise metric and 1,000 permutations against C2, C5, C6, and hallmark MsigDB. Reactome analysis was performed with PANTHER (Mi et al., 2013) using the “Statistical overrepresentation test” tool with default settings. Genes were considered significantly downregulated for reactome analysis when Log2FC > 2.5 and FDR *p* value < 0.05.

#### Analysis of Publicly Available Data

All publicly available data and software used for this publication are listed (Supplementary Material Table). OncoPrints were generated using cBioPortal (Gao et al., 2013). Briefly, OncoPrints generate graphical representations of genomic alterations, somatic mutations, copy number alterations, and mRNA expression changes. TCGA data was used for the different analyses.

## Data Availability Statement

The datasets presented in this study can be found in online repositories. The names of the repository/repositories and accession number(s) can be found below: https://www.ncbi.nlm.nih.gov/geo/, GSE163087.

## Ethics Statement

The animal study was reviewed and approved by the Regierung Unterfranken and the Ethics Committee under the license numbers 2532-2-362, 2532-2-367, 2532-2-374, and 2532-2-1003.

## Author Contributions

OH and MD: conceptualization. OH (*in vitro*), AB, JS, MR (RNA-Seq), CP-G (Biochemistry), CM, and WS (Mass Spec): methodology. MR (Bioinformatics), OH, MC, MTR, and MD (Pathology): formal analysis. OH, MR, TF, NP, MTR, and MD: investigation. AO, CD, MC, FB, MTR, and MD: resources. MD: writing—original draft. OH, MR, MTR, CD, AO, AS, and MD: writing—review and editing. MD: supervision and funding acquisition.

## Conflict of Interest

The authors declare that the research was conducted in the absence of any commercial or financial relationships that could be construed as a potential conflict of interest.

## Footnotes

1 https://qupath.github.io

2 https://doi.org/10.1038/s41598-017-17204-5

3 https://qupath.readthedocs.io/en/latest/

4 https://zlab.bio/guide-design-resources
