# Supplementary figures and images for: Implementation of CRISPR/Cas9 Genome Editing to Generate Murine Lung Cancer Models That Depict the Mutational Landscape of Human Disease
Source: Front Cell Dev Biol. 2021 Mar 2;9:641618. doi: 10.3389/fcell.2021.641618 (PMC7961101; doi:10.3389/fcell.2021.641618)

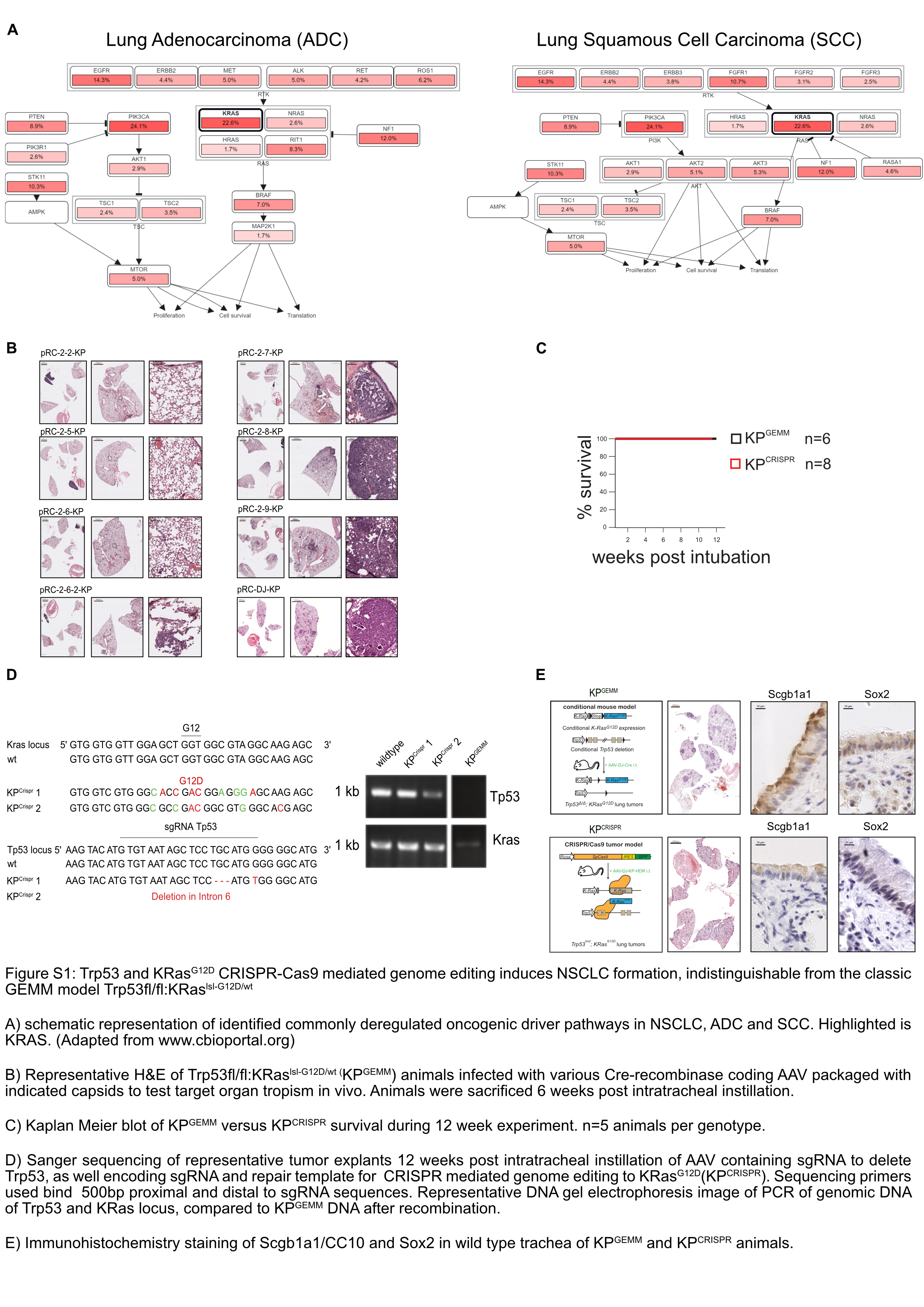

Supplement: Supplementary file 1 [file Image_1.tiff]

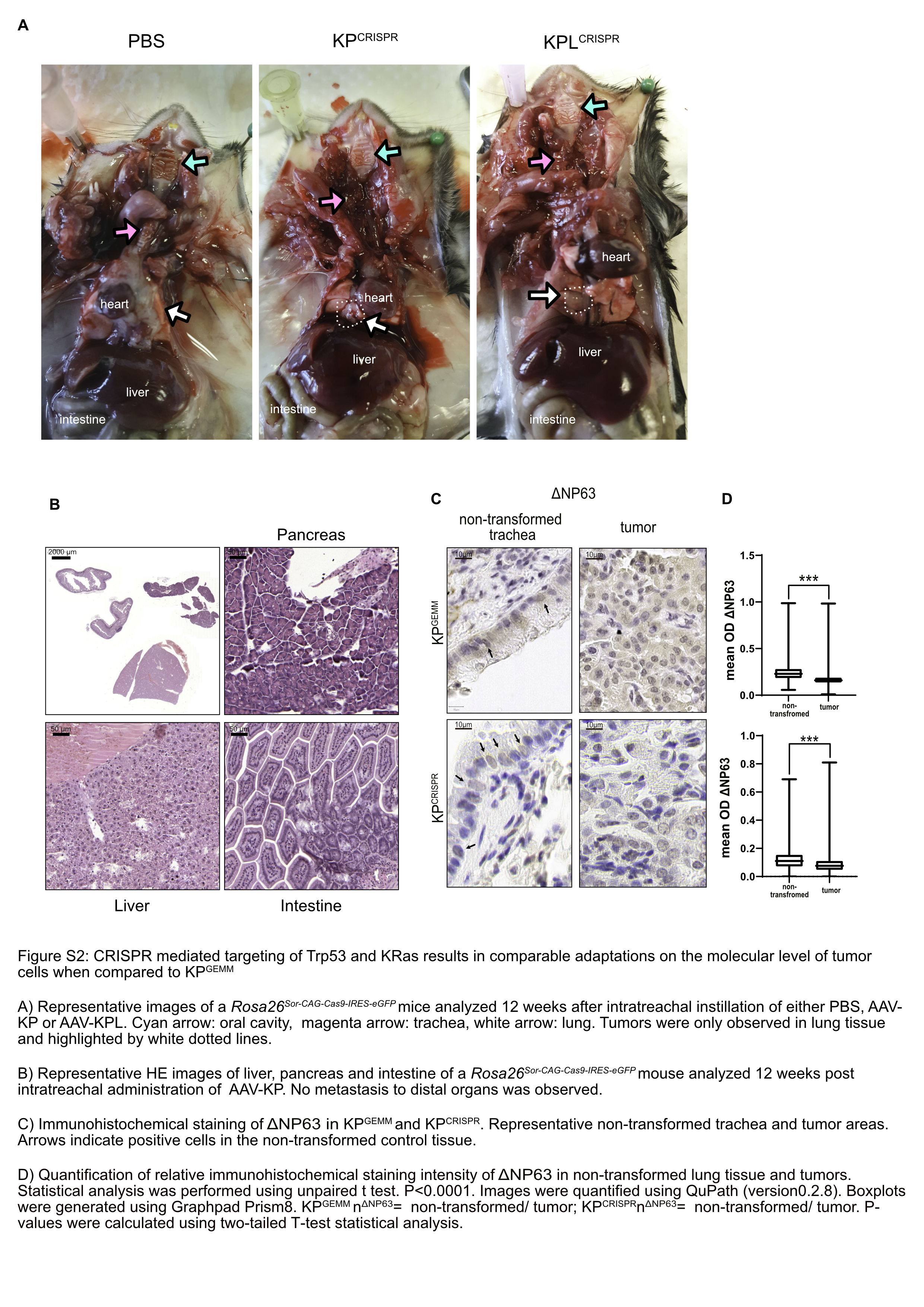

Supplement: Supplementary file 2 [file Image_2.tiff]

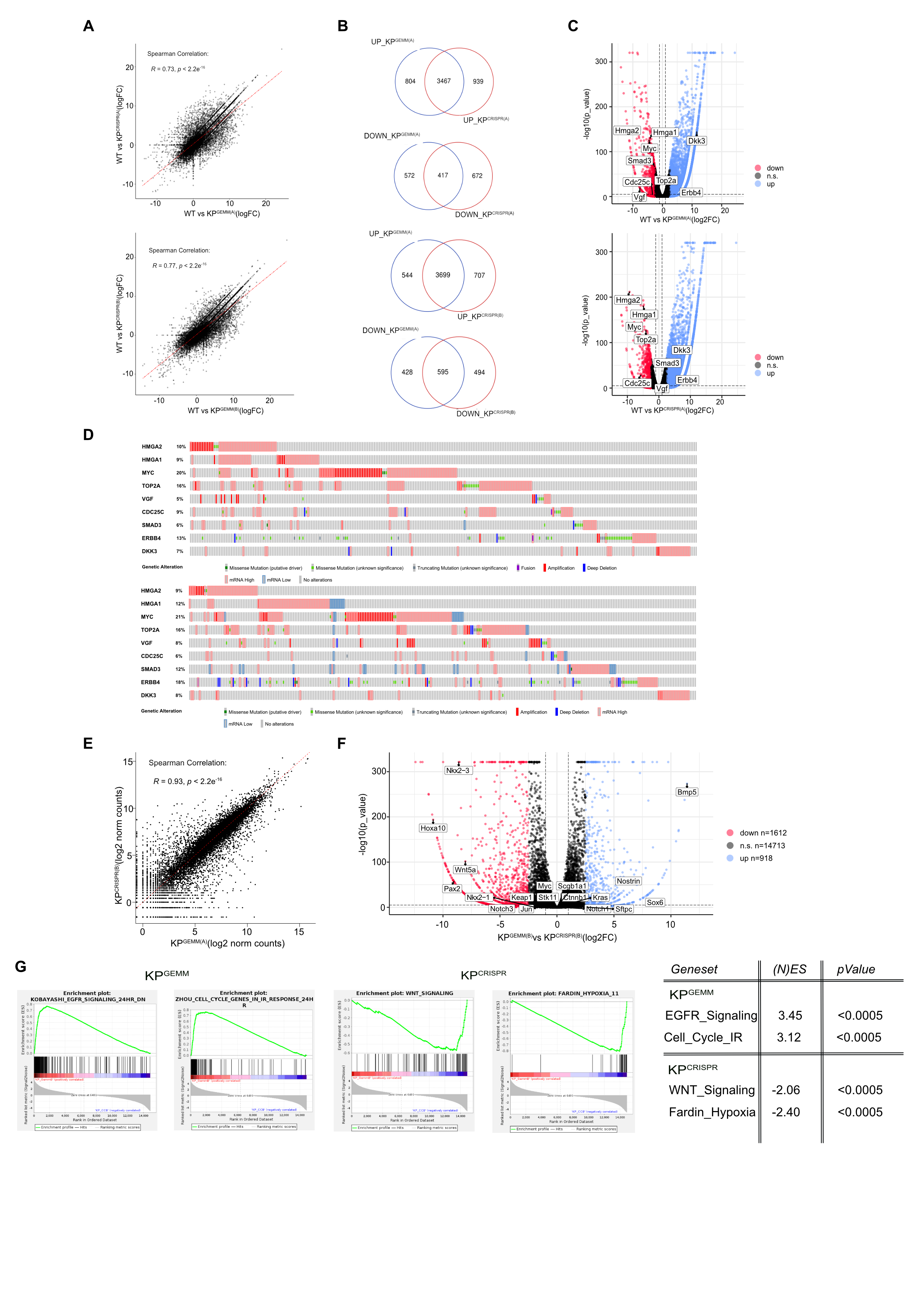

Supplement: Supplementary file 3 [file Image_3.tiff]

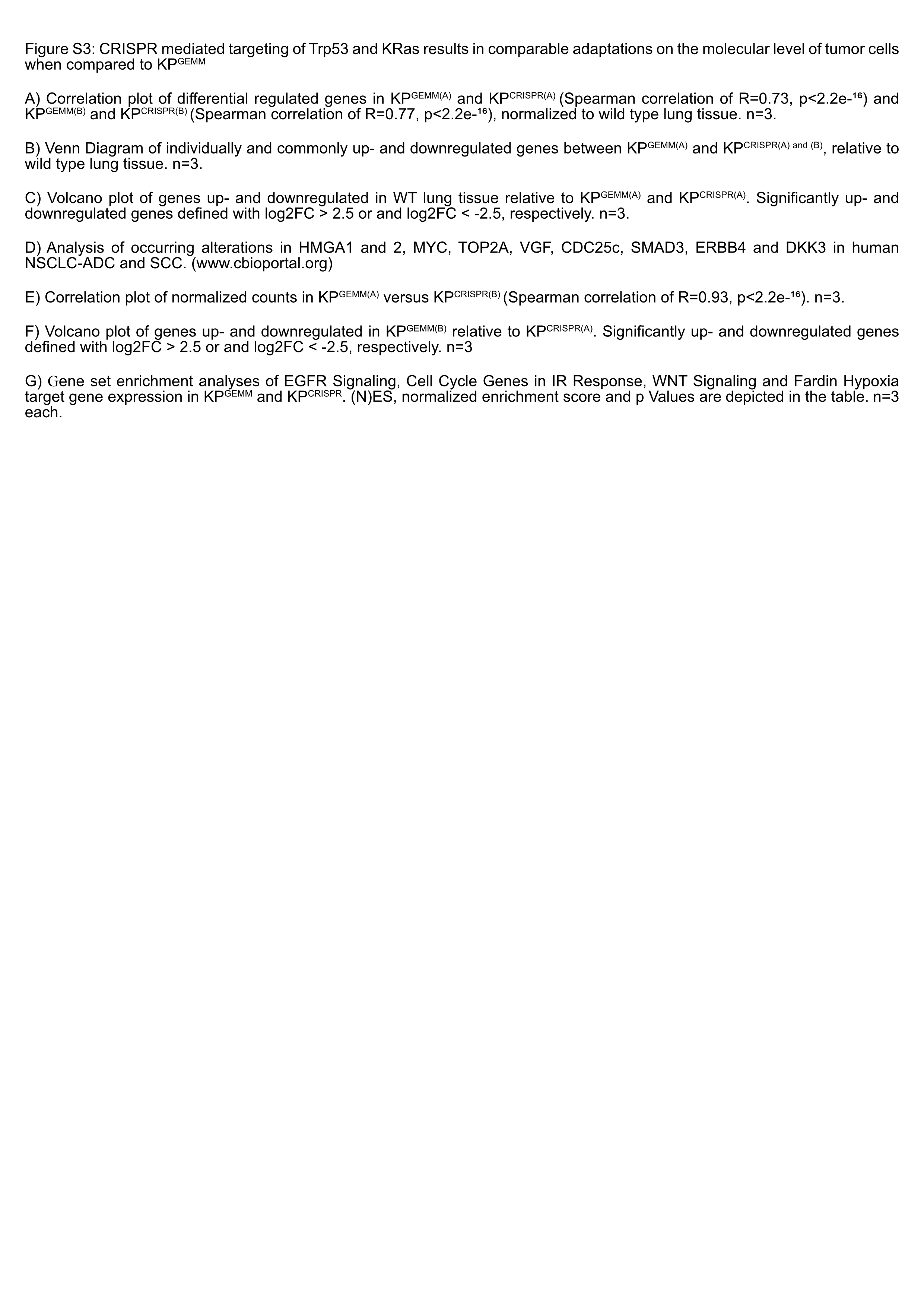

Supplement: Supplementary file 4 [file Image_4.tiff]

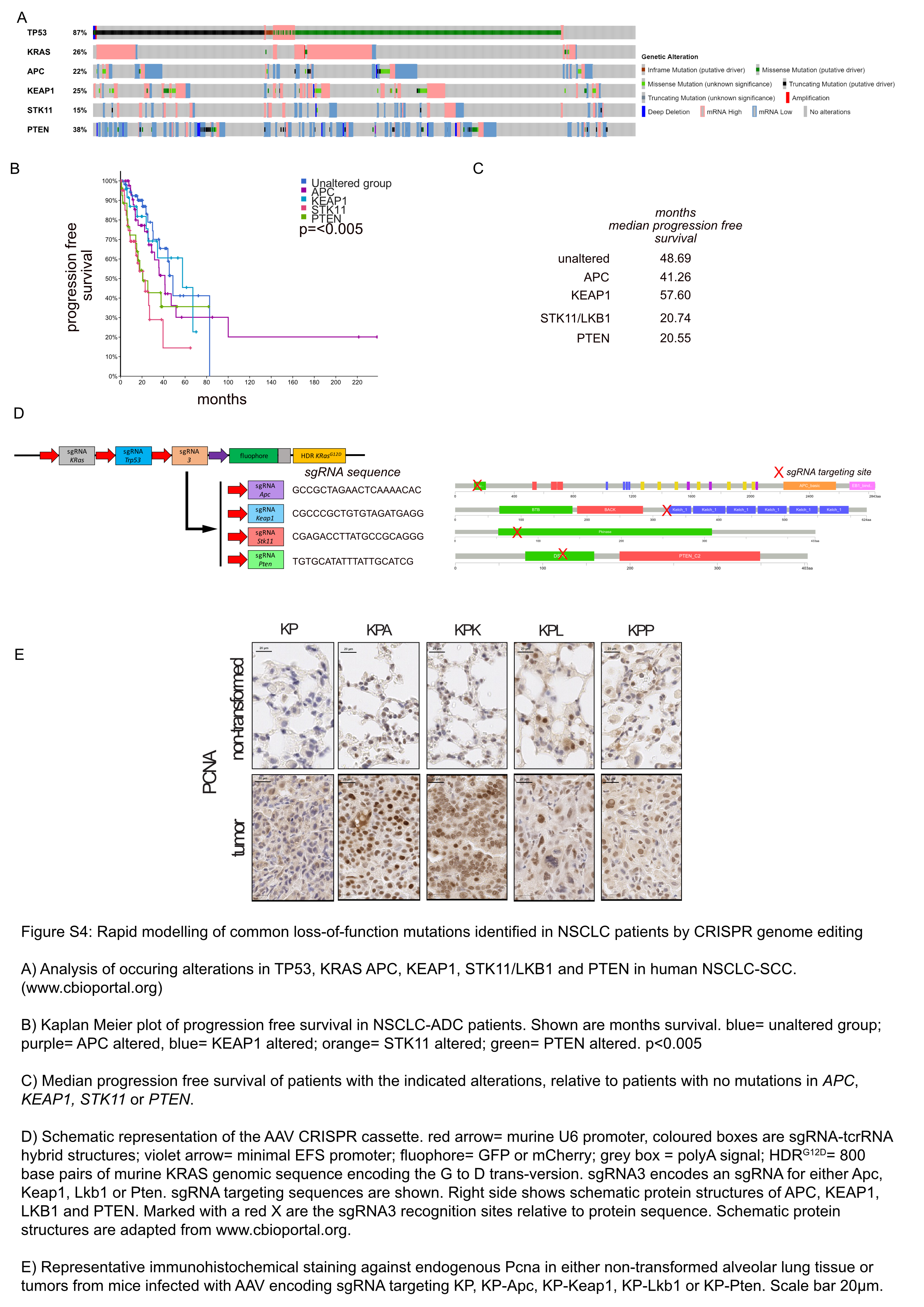

Supplement: Supplementary file 5 [file Image_5.tiff]

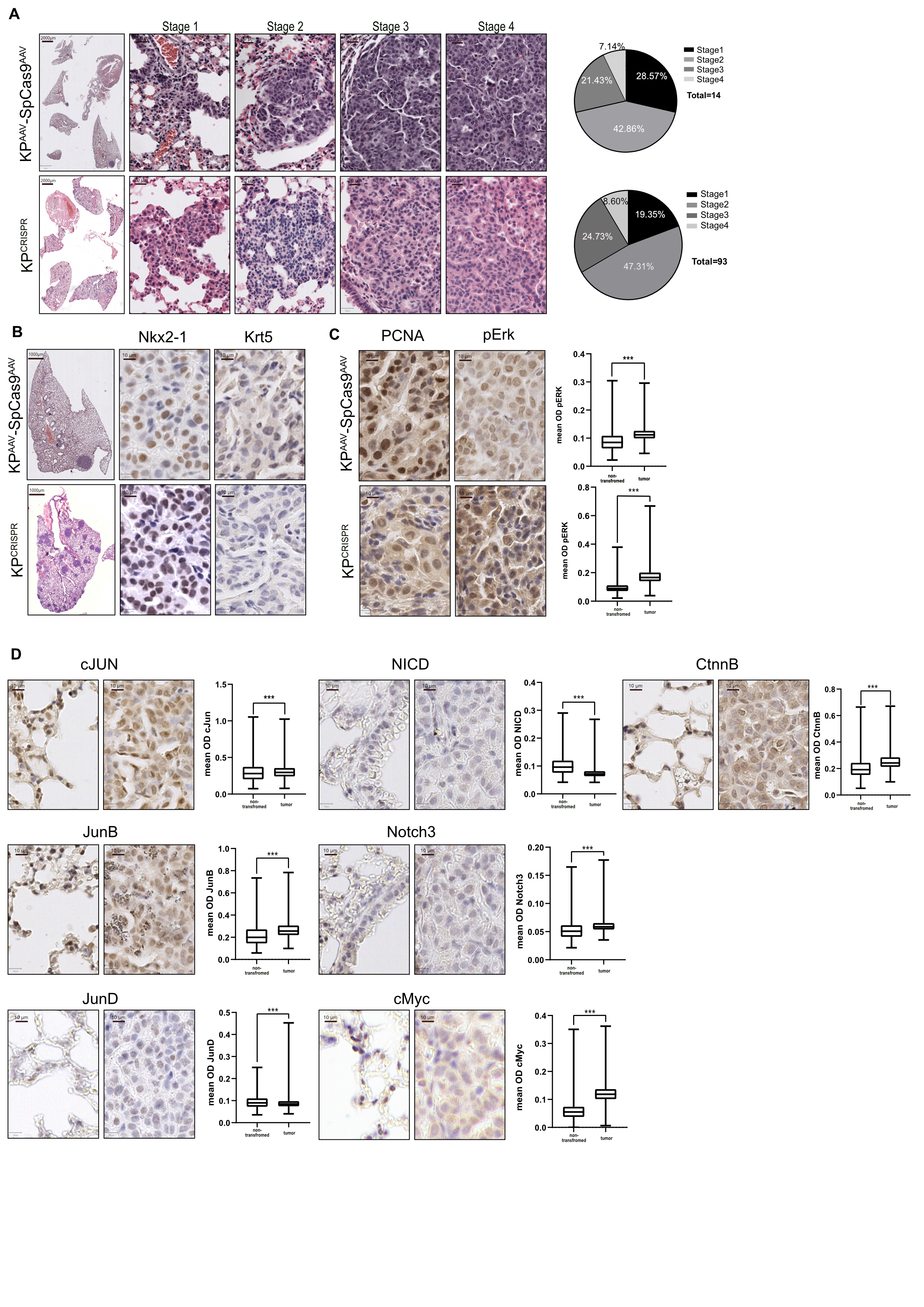

Supplement: Supplementary file 6 [file Image_6.tiff]

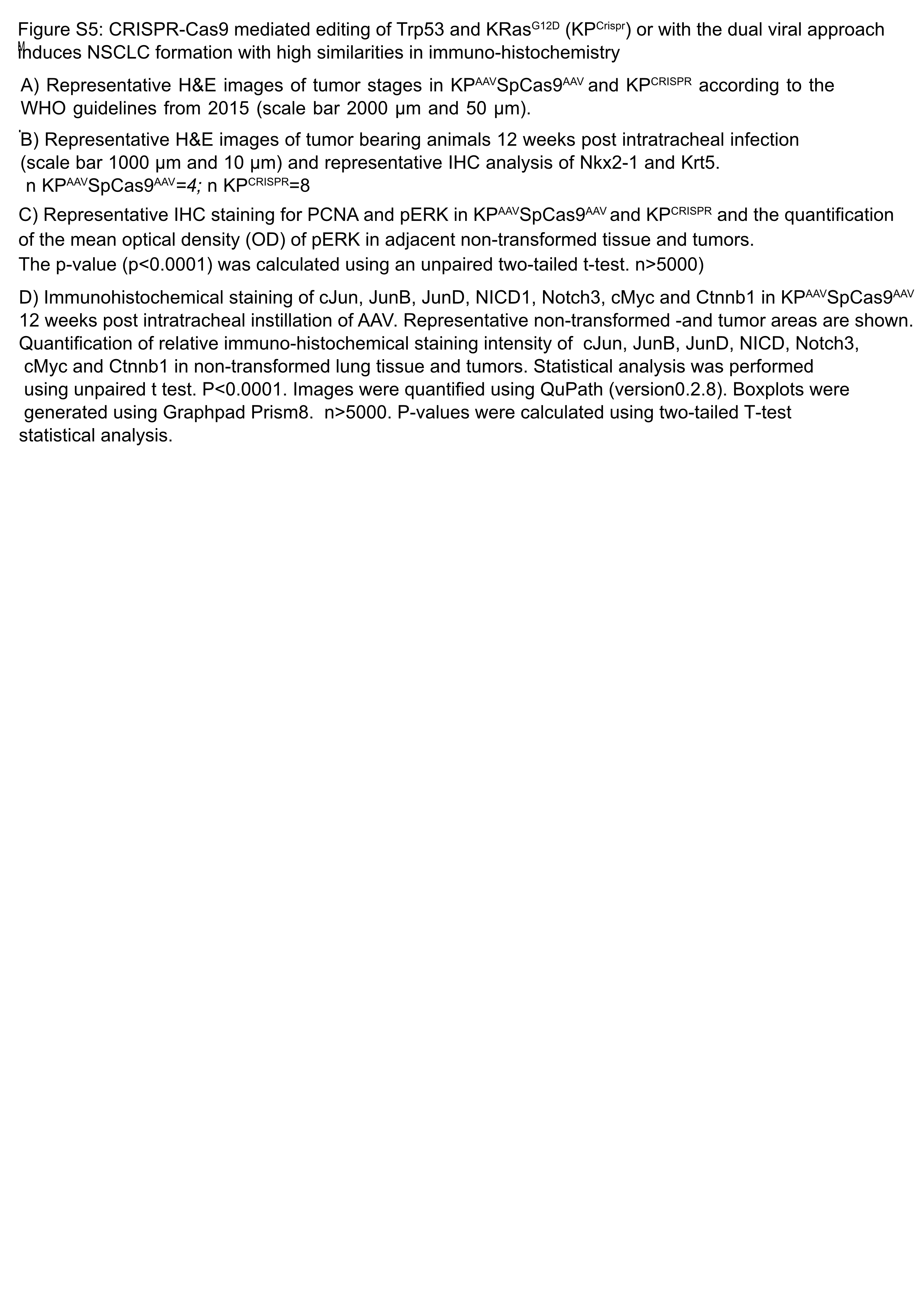

Supplement: Supplementary file 7 [file Image_7.tiff]
