# Supplementary material for: Implementation of CRISPR/Cas9 Genome Editing to Generate Murine Lung Cancer Models That Depict the Mutational Landscape of Human Disease
Source: Front Cell Dev Biol. 2021 Mar 2;9:641618. doi: 10.3389/fcell.2021.641618 (PMC7961101; doi:10.3389/fcell.2021.641618)
Supplement: Supplementary file 8 [file Table_1.DOCX]

| **1^st^ Antibodies** | **Company** | **Identifier** | **RRID** |
| --- | --- | --- | --- |
| Monoclonal rabbit anti-KRT5 recombinant mAb | Bimake | A5439 |  |
| Polyclonal rabbit anti-TTF1 (H-190) | Santa Cruz | sc-13040 | AB_793532 |
| Polyclonal rabbit anti-TP63 | Biolegend | 619002 | AB_2207170 |
| PCNA Antikörper (PC10) | Santa Cruz | Sc-56 | AB_628110 |
| p-ERK (E-4) | Santa Cruz | sc-7383 Lot: # L1714 | AB_627545 |
| CC10/Scgba1a1 | Proteintech Europe / PTGlab | 10490-1-AP | AB_2183285 |
| SFTPC | Proteintech Europe / PTGlab | 10774-1-AP | AB_2185497 |
| Sox2 | Sino Biological | 101284-T42 | AB_2810307 |
| anti-p63 (4A4) | Ventana | Cat# 790-4509 | AB_2335989 |
| NICD1 (active Notch1) | Abcam | ab8925 | AB_306863 |
| Notch 3 | Proteintech Europe / PTGlab | 55114-1-AP | AB_10858393 |
| Anti-Jun Clone 3/Jun (RUO) (8159868) | BD Biosciences | 610327 | AB_397717 |
| JunD | Sigma | HPA063029 | AB_2684925 |
| JunB | Santa Cruz | sc-8051 | AB_2130023 |
| mouse anti beta-Catenin | BD Biosciences | 610153 | AB_397554 |
| c-Myc (Y69) | Abcam | ab32072 | AB_731658 |
| APC | Sigma | HPA013349 | AB_1844913 |
| Keap1 | Proteintech Europe / PTGlab | 10503-2-AP | AB_2132625 |
| Lkb1/Stk11 | Proteintech Europe / PTGlab | 10746-1-ap | AB_2271311 |
| Pten | Proteintech Europe / PTGlab | 10047-1-AP | AB_2174343 |
| NFE2l2/Nrf2 | Invitrogen | PA5-27882 | AB_2545358 |
| Cas9 (bD-20) antibody - Lot: L0314 | Santa Cruz | sc-392737 |  |

**Consumables and Resources:**

| **­2^nd^ Antibodies** | **Company** | **Identifier** | **RRID** |
| --- | --- | --- | --- |
| SuperBoost™ Goat anti-Mouse Poly HRP | ThermoFisher | B40961 |  |
| SuperBoost™ Goat anti-Rabbit Poly HRP | ThermoFisher | B40962 |  |

| **Bacterial Strains** | **Company** | **Identifier** |
| --- | --- | --- |
| **DH5α** F- endA1 glnV44 thi-1 recA1 relA1 gyrA96 deoR nupG Φ80dlacZΔM15 Δ(lacZYA-argF)U169, hsdR17(rK-mK+), λ– | ThermoFisher | 18263012 |
| **Chemicals and Commercial Assays** | **Company** | **Identifier** |
| Gibco™  Dulbecco’s Modified Eagle Medium (DMEM), high glucose | ThermoFisher | 11574486 |
| Gibco™ RPMI 1640 Medium | ThermoFisher | 21875158 |
| Gibco™ Trypsin-EDTA (0.5%), No Phenol Red | ThermoFisher | 15400054 |
| Fetal Bovine Serum (FCS) | Sigma-Aldrich | 12103C |
| Penicillin-Streptomycin | Sigma-Aldrich | P4333 |
| Polybrene | Sigma-Aldrich | TR-1003 |
| Polyethylenimine, Linear, MW 25000, Transfection Grade (PEI 25K) | Polysciences | 23966-1 |
| Dimethyl sulfoxide (DMSO) | Sigma-Aldrich | D8418 |
| Ethanol (Etoh) | Carl Roth | 5054.6 |
| Gibco™ Phosphate-buffered saline (PBS) | ThermoFisher | 10010031 |
| Nuclease-free water | Merck | 3098 |
| Pierce™ Protein A/G Magnetic Beads | ThermoFisher | 88802 |
| Phosphate-buffered saline (PBS) | Homemade |  |
| 2-Propanol/ Isopropanol | ROTH | AE73.2 |
| Adenosintriphosphat (ATP) | Jena Bioscience | NU-1010-10G |
| Agarose | ROTH | 3810.4 |
| Ampicillin (Amp) | ROTH | HP62.2 |
| Bovine serum albumine (BSA) | Merck Millipore | 810683 |
| Dithiothreitol (DTT) | Sigma-Aldrich | D9779 |
| Eosin | Sigma | E4009 |
| Hematoxylin | Sigma | H3136 |
| Polyvinylidene difluoride membranes (PVDF) Immobilon Transfer Membrane | Merck | IPFL00010 |
| N,N,N',N'-tetramethylenethylendiamine (TEMED) | ROTH | 2367.3 |
| Natrium chloride (NaCl) | AppliChem | A2942,1000 |
| Neutrally buffered formalin (NBF) | Thermo Fisher | 5700TS |
| Tris-HCl | ROTH | 9090.5 |
| TritonX100 | ROTH | 3051.3 |
| Xylene | Sigma | 534056 |
| β-Mercaptoethanol | ROTH | 4227.1 |
| Methanol (MeOH) | ROTH | 0082.3 |
| Random Hexamer Primer | ThermoFisher | SO142 |
| RiboLock RNase Inhibitor | ThermoFisher | EO0381 |
| ReliaPrep™ RNA Cell Miniprep System Protocol | Promega | TM370 |
| NEBNext® Ultra™ II Directional RNA Library Prep Kit for Illumina® | New England Biolabs (NEB) | Cat #E7760L; Lot: 10065726 |
| NEBNext® Multiplex Oligos for Illumina® (Dual Index Primers Set 1) | New England Biolabs (NEB) | NEB #E7600S |
| NEBNext® Poly(A) mRNA Magnetic Isolation Module | New England Biolabs (NEB) | NEB #E7490S |
| Absource | Hyperactive In-Situ ChIP Library Prep Kit for Illumina® (pG-Tn5) | TD901-01 |
| Absource | VAHTS Universal Plus DNA Library Prep Kit for Illumina | ND617-01 |
| Absource | VAHTS Universal V6 RNA-seq Library Prep Kit for Illumina® | NR604-01 |
| Absource | VAHTS mRNA Capture Beads | N401-01 |
| Absource | VAHTS DNA Clean Beads | N411-02 |
| Absource | TruePrep DNA Library Prep Kit V2 for Illumina® (5 ng) | TD502-01 |
| NEBNext® Sample Purification Beads | New England BioLabs® Inc | Cat #E7767S; Lot: 10058432 |
| Histo-Clear^®^ Histological Clearing Agent | National Diagnostics | HS-200; Lot03-19-18 |
| Cytoseal 60^™^ | Thermo Scientific | 8310-4 |
| SignalStainR DAB Substrate Kit | Cell Signaling | 8059 S |
| **Cell lines**  **Cell** | **Company** | **Identifier** |
| Mouse: KP | Primary tumours |  |
| Mouse: KPA9 | Primary tumours |  |
| Mouse: KPK | Primary tumours |  |
| Mouse: KPL | Primary tumours |  |
| Mouse: KPP | Primary tumours |  |
| **Experimental Models: Organisms/Strains Cell** | **Company** | **Identifier** |
| B6(C)-Gt(ROSA)26Sor^em1.1(CAG-cas9*,-EGFP)Rsky^/J | The Jackson laboratory | Stock No: 028555 |
| B6.129-Kras^tm4Tyj^ Trp53^tm1Brn^/J | The Jackson laboratory | Stock No: 032435 |
| C57BL/6J | The Jackson Laboratory | Stock No: 000664 |
| **Oligonucleotides** | **Sequence** | **Company** |
| sgRNA murine Stk11/Lkb1 for | CACCGCGAGACCTTATGCCGCAGGG | Sigma |
| sgRNA murine Stk11/Lkb1 rev | AAACCCCTGCGGCATAAGGTCTCGC | Sigma |
| sgRNA murine APCex9 for | CACCGCCGCTAGAACTCAAAACAC | Sigma |
| sgRNA murine APCex9 rev | AAACGTGTTTTGAGTTCTAGCGGC | Sigma |
| sgRNA murine KEAP1 for | CACCGCGCCCGCTGTGTAGATGAGG | Sigma |
| sgRNA murine KEAP1 rev | AAACCCTCATCTACACAGCGGGCGC | Sigma |
| sgRNA murine Pten 1 for | CACCGTGTGCATATTTATTGCATCG | Sigma |
| sgRNA murine Pten 1 rev | AAACCGATGCAATAAATATGCACAC | Sigma |
| sgRNA murine Kras #1 for | CACCGACTGAGTATAAACTTGTGG | Sigma |
| sgRNA murine Kras #1 rev | AAACCCACAAGTTTATACTCAGTC | Sigma |
| sgRNA murine Trp53 #1 for | CACCGATGGTGGTATACTCAGAGC | Sigma |
| sgRNA murine Trp53 #1 rev | AAACGCTCTGAGTATACCACCATC | Sigma |
| KrasG12D repair template for | TTTTGTGTAAGCTTTGGTAACTCCATGTATTTTTATTAAGTGTT | Sigma |
| KrasG12D repair template rev | GAGCTTATCGATACCGTCGACACACCCAGTTTAAAGCCTTGGAA | Sigma |
| **Recombinant DNA** | **Company/Source** | **Identifier** |
| pHelper | Cell Biolabs, INC. | VPK-400-DJ |
| pAAV-DJ Vector | Cell Biolabs, INC. | VPK-420-DJ |
| AAV:ITR-U6-sgRNA(Kras)-U6-sgRNA(p53)-pEFS-2A-mCherry-shortPA-KrasG12D_HDRdonor-ITR | doi: 10.15252/emmm.201911101 | N/A |
| AAV:ITR-U6-sgRNA(Kras)-U6-sgRNA(p53)-U6-sgRNA(Lkb1)- pEFS-2A-mCherry-shortPA-KrasG12D_HDRdonor-ITR | doi: 10.15252/emmm.201911101 | N/A |
| AAV:ITR-U6-sgRNA(Kras)-U6-sgRNA(p53)-U6-sgRNA(Lkb1)-U6-sgRNA(Pten)-pEFS-2A-mCherry-shortPA-KrasG12D_HDRdonor-ITR | This publication | N/A |
| AAV:ITR-U6-sgRNA(Kras)-U6-sgRNA(p53)-U6-sgRNA(Lkb1)-U6-sgRNA(Apc-Exon9)-pEFS-2A-mCherry-shortPA-KrasG12D_HDRdonor-ITR | This publication | N/A |
| AAV:ITR-U6-sgRNA(Kras)-U6-sgRNA(p53)-U6-sgRNA(Lkb1)-U6-sgRNA(Keap1)-pEFS-2A-mCherry-shortPA-KrasG12D_HDRdonor-ITR | This publication | N/A |
| AAV:ITR-U6-sgRNA(Kras)-U6-sgRNA(p53)-U6-sgRNA(Lkb1)-pEFS-Rluc-2A-Cre-shortPA-KrasG12D_HDRdonor-ITR (AAV-KPL) | AAV:ITR-U6-sgRNA(Kras)-U6-sgRNA(p53)-U6-sgRNA(Lkb1)-pEFS-Rluc-2A-Cre-shortPA-KrasG12D_HDRdonor-ITR (AAV-KPL) was a gift from Feng Zhang (Addgene plasmid # 60224 ; http://n2t.net/addgene:60224 ; RRID:Addgene_60224) | Addgene plasmid # 60224 |
| **Software and Algorithm** | **Company/Source** |  |
| cBioportal | https://www.cbioportal.org |  |
| GEPIA | http://gepia.cancer-pku.cn |  |
| KM-plotter | http://kmplot.com/analysis/ |  |
| BoxPlotR | http://shiny.chemgrid.org/boxplotr/ |  |
| Excel | Microsoft |  |
| Venn diagrams | http://bioinformatics.psb.ugent.be/webtools/Venn/ |  |
| Image Studio | Licor |  |
| Panther Classification system | http://pantherdb.org |  |
| Nemates | http://nemates.org |  |
| GraphPad Software | GraphPad Software, Inc. |  |
| Affinity Designer | Serif Europe |  |
| ImageJ | National Insistute of Health |  |
| Primerx | http://www.bioinformatics.org/primerx/cgi-bin/DNA_1.cgi |  |
| Pannoramic Case Viewer | 3dHistech |  |
| R2: Genomics Analysis and Visualization Platform | http://r2.amc.nl |  |
| GenerateFastq v1.1.0.64 | http://emea.support.illumina.com/downloads/local-run-manager-generate-fastq-module.html |  |
| FastQC | http://www.bioinformatics.babraham.ac.uk/projects/fastqc/ |  |
| Bowtie2 v2.3.4.1 | http://bowtie-bio.sourceforge.net/index.shtml |  |
| TopHat v.2.1.1 | <https://ccb.jhu.edu/software/tophat/index.shtml> |  |
| Samtools v1.3 | http://samtools.sourceforge.net |  |
| R | https://www.r-project.org |  |
| EdgeR | <https://bioconductor.org/packages/release/bioc/html/edgeR.html> |  |
| GenomicAlignments | <https://bioconductor.org/packages/release/bioc/html/GenomicAlignments.html> |  |
| GSEA v2.2 | <http://software.broadinstitute.org/gsea/downloads.jsp> |  |
| QuPath Version: 0.2.3 | Bankhead, P. et al. (2017). QuPath: Open source software for digital pathology image analysis. Scientific Reports. https://doi.org/10.1038/s41598-017-17204-5 |  |
| **Instrument** | **Company/Source** |  |
| StepOnePlus Real-Time PCR System | ThermoFisher |  |
| Invitrogen Countess II FL Automated Cell Counter | ThermoFisher |  |
| Pannoramic DESK scanner DW II | 3DHISTECH |  |
| Ventana DP200 | Roche |  |
| FSX100 microscopy | Olympus Life Science |  |
| Fragment Analyzer | Agilent formerly Advanced Analytical |  |
| Axiocam 503 mono + Zeiss axio microscope | Zeiss |  |
| Branson Sonifier 250 | Branson |  |
| Hyrax M55 Rotary Microtome | Leica |  |
| PCR cycler: SimpliAmp thermo cycler | Life technologies |  |
